# Cytochrome P450 enzyme-mediated auto-enhanced photodynamic cancer therapy of co-nanoassembly between clopidogrel and photosensitizer

**DOI:** 10.7150/thno.42633

**Published:** 2020-04-15

**Authors:** Qiu Wang, Mengchi Sun, Dan Li, Chang Li, Cong Luo, Zhaomeng Wang, Wenjuan Zhang, Zimeng Yang, Yao Feng, Shuang Wang, Zhonggui He, Haotian Zhang, Qiming Kan, Wei Sun, Jin Sun

**Affiliations:** 1 Wuya College of Innovation, Shenyang Pharmaceutical University, Shenyang, 110016, P. R. China; 2 Key Laboratory of Structure-Based Drug Design and Discovery, Ministry of Education, Shenyang Pharmaceutical University, Shenyang, 110016, P. R. China; 3 School of Pharmacy, Shenyang Pharmaceutical University, Shenyang, 110016, P. R. China; 4 School of Life Science and Biopharmaceutics, Shenyang Pharmaceutical University, Shenyang, 110016, P. R. China; 5 College of Medical Device, Shenyang Pharmaceutical University, Shenyang, 110016, P. R. China

**Keywords:** cytochrome P450 enzyme, glutathione, photodynamic therapy, clopidogrel, disrupt intracellular redox homeostasis

## Abstract

Reactive oxygen species (ROS)-based photodynamic therapy (PDT) has a widespread application in cancer therapy. Nevertheless, the efficiency of PDT is far from satisfactory. One major impediment is the overexpression of glutathione (GSH) in tumor cells, which could deplete the level of PDT-generated ROS. Herein, we develop a novel type of cytochrome P450 enzyme-mediated auto-enhanced photodynamic co-nanoassembly between clopidogrel (CPG) and photosensitizer pyropheophorbide a (PPa).

**Methods:** In this work, we prepare the co-assembled nanoparticles of CPG and PPa (CPG/PPa NPs) by using one-step precipitation method. The assembly mechanism, drug release behavior, GSH consumption, ROS generation, cellular uptake, cytotoxicity of CPG/PPa NPs are investigated *in vitro*. The mice bearing 4T1 tumor are employed to evaluate *in vivo* biodistribution and anti-tumor effect of CPG/PPa NPs.

**Results:** Such CPG/PPa NPs could disrupt the intracellular redox homeostasis, resulting from the elimination of GSH by CPG active metabolite mediated by cytochrome P450 enzyme (CYP2C19). The *in vivo* assays reveal that CPG/PPa NPs not only increase the drug accumulation in tumor sites but also significantly suppress tumor growth in BALB/c mice bearing 4T1 tumor. With CPG-mediated GSH consumption and PPa-triggered ROS generation, CPG/PPa NPs show the enhanced PDT treatment effect by breaking intracellular redox balance.

**Conclusion:** Our findings provide a valuable knowledge for the rational design of the PDT-based combinational cancer therapy.

## Introduction

Photodynamic therapy (PDT) has been an appealing cancer treatment strategy due to its specific spatiotemporal selectivity, non-invasiveness, and less undesired side effects, compared to traditional chemotherapy strategies 1-6. The efficacy of PDT strongly depends on the level of reactive oxygen species (ROS), such as singlet oxygen (^1^O_2_) generated by photosensitizers (PSs) upon specific laser irradiation to induce tumor cells necrosis or apoptosis 7-13. However, tumor cells themselves exist various defense systems (e.g., up-regulating antioxidants or enzymes) to hold redox homeostasis for cell survival, which impedes oxidative damage and then reduces apoptosis of cancer cells during the PDT process 14-16.

Glutathione (GSH) plays a crucial role in maintaining redox homeostasis of cells, and is overexpressed in cancer cells compared to normal cells 17-20. Furthermore, GSH, an important scavenger of ROS, could compromise ROS-based cancer therapy such as PDT and radiotherapy 21-23. Hence, great efforts have been devoted to down-regulate the level of GSH in order to achieve the desired therapeutic effect. For instance, Liu's group employed L-buthionine sulfoximine (a GSH inhibitor) to inhibit γ-glutamylcysteine synthetase, thus improving the therapeutic effect by increasing tumor cellular oxidative stress 15. Besides, several nanomaterials such as MnO_2_ nanosheets 24 and copper (II)-graphitic carbon nitride 25,26, have been used to consume GSH. Although these strategies promote the anticancer effects of ROS-based cancer therapy, there still exist of several restrictions. L-buthionine sulfoximine (BSO) has high oxidative stress to normal cells 27,28, and reduction products of MnO_2_ and copper (II) (Mn (II) and Cu(I)) have potentially toxic effects on the human body 29, thus hindering their further clinical translation. Therefore, exploitation of the GSH consumer activated by cytochrome P450 enzyme in tumor microenvironment for selective cytotoxicity is extremely significant.

Clopidogrel (CPG), a classical anti-platelet prodrug, is widely used in the treatment of thrombosis 30. It has been reported that cytochrome P450 (CYP2C19) enzyme mediates the metabolism of CPG to form thiol-containing metabolites 31. And the anti-platelet mechanism of CPG is relevant to depletion of intracellular GSH by formation of the disulfide bond between cysteine residues of GSH and the thiol-containing metabolites of CPG 32. Subsequently, the conjugate (CPG-SS-GSH) acts on adenosine diphosphate (ADP) receptor P2Y_12_ and inhibits ADP-mediated platelet aggregation 33. Inspired by the action mechanism of CPG, we hypothesize that CPG could strengthen the efficacy of PDT via consuming the intracellular GSH amount in CYP2C19-expressed cancer cells.

**Figure 1:**
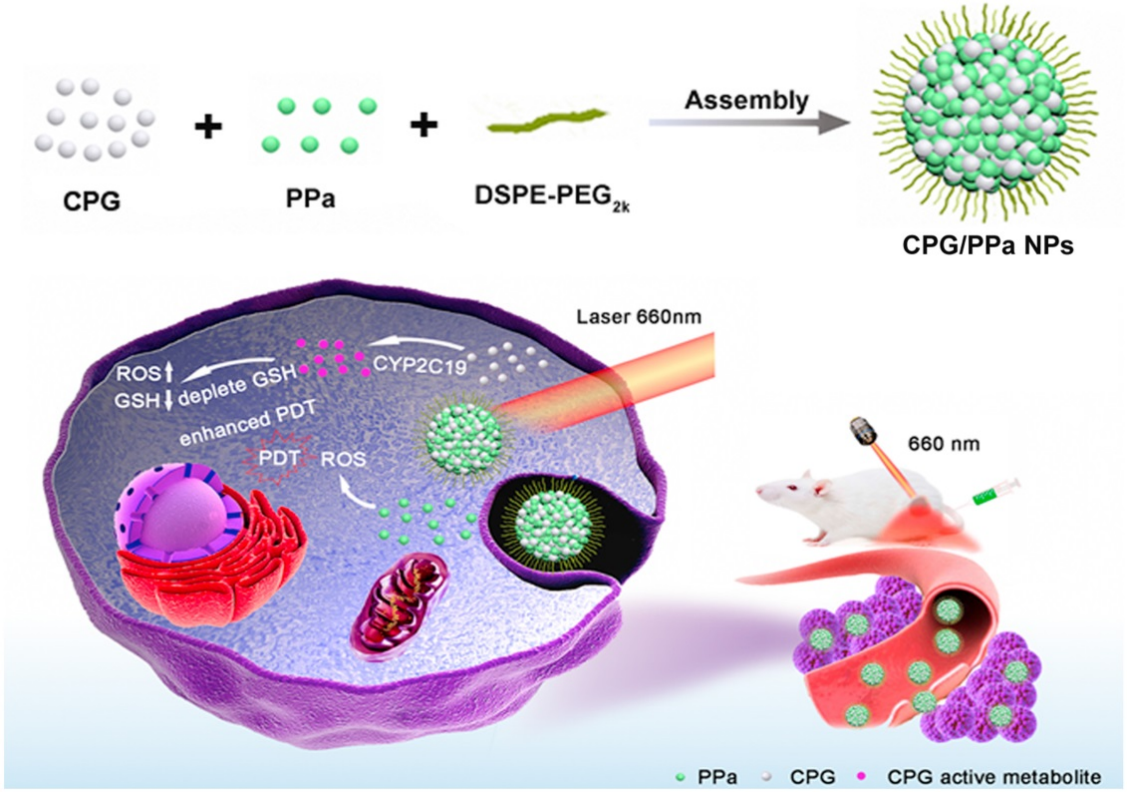
** Schematic representation the preparation process and synergistic antitumor effect of CPG/PPa NPs under laser irradiation.** The nanoassemblies were formed by CPG and PPa. The surface of nanoparticles was modified with DSPE-PEG_2k_ to improve the stability (CPG/PPa NPs). After delivery of CPG/PPa NPs to targeted tumor cells, CPG was bio-activated by CYP2C19, achieving auto-enhanced antitumor effect under laser irradiation.

Given that CYP2C19 could mediate GSH depletion by the thiol-containing metabolites of CPG, we firstly developed the self-delivery CPG and photosensitizer pyropheophorbide (PPa) co-assembly nanoparticles (CPG/PPa NPs) for auto-enhanced photodynamic therapy (Figure 1). Interestingly, CPG and PPa could form stable near-spherical nanostructures by strong π-π stacking, hydrophobic interactions, hydrogen bond, and electrostatic interactions confirmed by computational simulations and experimental study. CPG/PPa NPs exhibited higher cytotoxicity and greater accumulation at tumor sites compared to free PPa. More importantly, CPG/PPa NPs demonstrated great synergistic anti-tumor effects by depleting GSH to break the redox homeostasis of tumor cells, and then enhanced the efficacy of PDT in 4T1 breast tumor xenograft model. Such a unique metabolism-based combination and structure-based co-assembly nanosystem provides a novel platform for the combinational PDT in cancer therapy.

## Methods

### Materials

Pyropheophorbide a (PPa) was obtained from Shanghai Dibai Chemical Technology Co. Ltd. Clopidogrel bisulfate and Glutathione reduced ethyl ester (GSH-OEt) were purchased from Shanghai Aladdin Biochemical Technology Co., Ltd. 1,2-distearoylsn-glycero-3-phosphoethanolamine-N-[methoxy(polyethyleneglycol)-2000 (DSPE-PEG_2k_) was bought from Shanghai Advanced Vehicle Technology Co. Ltd, China. Cell culture reagents, intracellular ROS test kits (2,7-Dichlorodihydrofluorescein diacetate, DCFH-DA), 4',6-diamidino-2-phenylindole (DAPI), 3-(4,5-dimethyl-2-thiazolyl)-2,5-diphenyl-2-H-tetrazolium bromide (MTT), Trypsin-Ethylenediaminetetraacetic acid (trypsin-EDTA) were supplied by Dalian Meilun Biotech Co., Ltd., China. Test kits of CYP2C19 enzyme activity and content were obtained from Shanghai Yuanmu Biotechnology Co. Ltd, China. BCA protein kit was bought from Beyotime. GSH assay kit was purchased from Nanjing Jiancheng Bioengineering Institute. Other regents and chemicals applied in the article were of analytical standard grade.

### Cell culture

Mouse prostatic carcinoma cells (RM-1) and mouse breast carcinoma cells (4T1) were maintained with Roswell Park Memorial Institute (RPMI 1640) medium including streptomycin (100 μg/mL), 10% fetal bovine serum (FBS), and penicillin (100 units/mL). Human liver carcinoma cells (HepG2) and human normal liver cells (L02) were cultured in Dulbecco's Modified Eagle Media (DMEM) including the same reagents as mentioned above. The culture conditions of all cells were humidified atmosphere of 5% CO_2_ at 37 °C.

### Synergistic effects of CPG and PPa at various combination ratios

Synergy of PPa in combination with CPG was determined by utilizing MTT method. 4T1 cells were seeded into 96-well cell-culture plates (2 × 10^3^ cells/well). After 12 h incubation, cells were exposed to CPG, PPa or mixture of CPG and PPa (CPG/PPa) at a molar of 5:1, 2:1, 1:1, 1:2 and 1:5. Cells were cultured with these drugs for 4 h, then they were irradiated with laser (660 nm, 30 mW/cm^2^) for 2 min. After that, these 4T1 cells were further cultured until 48 h before MTT determination. The synergistic effect of CPG/PPa was estimated via calculating the combination index (CI). CI of CPG and PPa was calculated in terms of the following equation expressed by Gao 34:

CI=IC_50_ (CPG in PPa)/IC_50_ (CPG) + IC_50_ (PPa in CPG)/IC_50_ (PPa)

IC_50_ (CPG) was used to calculate the IC_50_ when CPG was administrated separately. IC_50_ (CPG in PPa) was used to calculate the IC_50_ of CPG when CPG and PPa were administrated collectively; IC_50_ (PPa) was used to calculate the IC_50_ when PPa was administrated separately; IC_50_ (PPa in CPG) was used to calculate the IC_50_ of PPa when CPG and PPa were administrated collectively. The classifications of synergy are additivity (CI=1), synergistic effect (CI < 1), or antagonistic effect (CI > 1).

### The preparation and characterization of non-PEGylated CPG/PPa NPs

Non-PEGylated CPG/PPa NPs were fabricated by a one-step precipitation method 35. In short, 4mg desalted CPG was dissolved in 2 mL methanol. 4 mg PPa was dissolved in mixed solvent (2 mL methanol/tetrahydrofuran=1:1, v/v) to acquire PPa solution; then the mixed solution of 240 μL CPG and 200 μL PPa was dripped into deionized aqueous solution (2 mL) and agitated for 20 minutes (900 rpm). Finally, organic solvent was removed under vacuum at 37 °C. The morphology, zeta potential and the size of non-PEGylated CPG/PPa NPs were characterized by a transmission electron microscopy (TEM) and Zetasizer, respectively. In addition, the colloidal stability of non-PEGylated incubated with PBS (pH 7.4) including 10% FBS for 4 h was investigated by monitoring the particle size of NPs.

### Construction of CPG/PPa NPs

To improve the stability of non-PEGylated CPG/ PPa NPs, DSPE-PEG_2k_ was used to modify the surface of NPs, obtaining CPG/PPa NPs. CPG/PPa NPs were fabricated in the same process (The preparation of non-PEGylated CPG/PPa NPs) using a mixed solution of CPG, PPa and DSPE-PEG_2k_ (20 wt%).

### Characterization of CPG/PPa NPs

The morphology, zeta potential and the size of CPG/PPa NPs were determined by a TEM and Zetasizer, respectively. Besides, the colloidal stability of CPG/PPa NPs was assessed by monitoring particle size change. CPG/PPa NPs were added to PBS (pH 7.4), RPMI 1640 and DMEM medium including 10% FBS, then the nanoparticles were incubated for 12 h in shaking table (37 °C). Additionally, the colloidal stability of CPG/PPa NPs in plasma was further investigated. The CPG/PPa NPs with PPa concentration of 200 µg/mL (1 mL) were incubated with rat plasma (100 μL) for 12 h at 37 °C 36. The change of CPG/PPa NPs size was monitored at predesigned timepoints (0, 2, 4, 6, 8, 10 and 12 h). The encapsulation efficiency (EE) and loading efficiency (LE) of CPG were determined by high-performance liquid chromatography (HPLC). The EE and LE of PPa were measured by using a microplate reader (excitation wavelength: 415 nm, emission wavelength: 675 nm). To investigate the interaction between CPG and PPa in CPG/PPa NPs, CPG/PPa NPs were treated with different concentrations of NaCl, SDS and urea. Additionally, the CPG/PPa NPs treated with NaCl, SDS, and urea (100 mM) were further characterized by TEM.

### Assembly simulation

In our study, computational simulations were employed to investigate the assembly mechanism between CPG and PPa molecules. And Sybyl software was applied to obtain the 3-dimentional structures of CPG and PPa. The optimized parameters and runtime environment were in accordance with our previous work 37. Discovery Studio 2017 Visualizer software was used to analyze the ultimate results.

### Ultraviolet and fluorescence spectra

Free PPa solution, free CPG solution, CPG/PPa NPs, sodium dodecyl sulfate (SDS), and CPG/PPa NPs (at a PPa equivalent of 10 ug/mL) containing SDS (0.2% w/v), the ultraviolet (UV) absorbance spectra of them were characterized by ultraviolet spectrophotometer (UV1102II). The PPa fluorescence spectra of PPa solution and CPG/PPa NPs (at a PPa equivalent concentration) were obtained by a multifunctional microplate reader.

### *In vitro* drug release profiles

For the experiment, dialysis method was conducted to evaluate the release behavior of CPG/ PPa NPs *in vitro*. PBS (pH 6.5 or 7.4) containing 15% tetrahydrofuran was selected as release medium. 1 mL CPG/PPa NPs suspension was added into a dialysis membrane, and immersed in conical flask including 30 mL release medium in shaking table (100 rpm) at 37 °C. At predesigned timepoints, 1mL release medium was taken out and 1mL medium was supplemented. The cumulative release of CPG and PPa from CPG/PPa NPs were determined by HPLC and microplate reader, respectively. The conditions of chromatographic separation were as follows: C_18_ chromatographic column (4.6 × 250 mm, 5 μm); Mobile phase A: acetonitrile, 70%; Mobile phase B: water containing 0.01 mol/L potassium dihydrogen phosphate, 30%. The flow rate was set to 1.0 mL/min, and CPG was detected at wavelength 220 nm. Besides, the stability of in CPG/PPa NPs in solution with pH 6.5 was further investigated. The CPG/PPa NPs were incubated with PBS (pH 6.5) for 4 h at 37 °C, the size change was assessed to the stability of NPs.

### Content and activity of CYP2C19 assay

4T1, RM-1, HepG2 and L02 cells (1 × 10^5^ cells/well) were cultured in 12-well cell-culture plates for 48 h. Afterwards, these cells were detached with trypsin and repeated freeze-thaw cycles three times. Then, the centrifugation (3000 rpm, 5 min) was carried out to collect the supernatants. The content and activity of CYP2C19 were assayed by Elisa Kit (Shanghai, China).

### Detection of cellular GSH

4T1, RM-1, HepG2 and L02 cells were cultured in 12-well cell-culture plates at a certain density of 3 × 10^5^ cells per well for 12 h. Subsequently, various concentrations of CPG/PPa NPs, CPG, and PPa were added to 12-well plates, and further incubated for 24 h. After incubation, the treated cells were repeated freeze-thaw cycles thrice with liquid nitrogen. Finally, the centrifugation (3000 rpm, 5 min) was carried out to harvest the supernatants. The GSH relative content of four cells were determined based on Reduced Glutathione Assay Kit (Nanjing, China).

### Intracellular uptake

4T1 cells (5 × 10^4^ cells/well) were seeded in 24- well plates and cultured for 12 h. Then medium was replaced by CPG/PPa NPs or free PPa (2.5 μg/mL), and incubated for 1 h, 2 h and 4 h. Afterwards, removing the medium and rinsing three times with ice-cold PBS, and then fixing with 4% paraformaldehyde. Subsequently, DAPI was added to the 24-well plates to stain the cell nuclei for 10 min. Finally, confocal laser scanning microscopy (CLSM) was applied to observe the fluorescence signals of PPa in 4T1 cells.

For intracellular quantitative uptake, 4T1 cells (5 × 10^5^ cells/well) were cultured in culture dishes for 48 h. Then discarding the medium, CPG/PPa NPs or free PPa (2.5 μg/mL) were added to culture dishes for 2 h and 4 h, respectively (n=3). Subsequently, cells in the dishes were rinsed with ice-cold PBS and collected. Finally, HPLC was used to analyze the intracellular content of PPa.

### Detection of cellular ROS

DCFH-DA was used to detect the ROS generation in 4T1 cells. DCFH-DA itself does not produce fluorescent signal, but it could generate fluorescent DCF when reacting with intracellular ROS. 4T1 cells (1 × 10^5^ cells/well) were seeded in 12-well cell-culture plates and cultured for 12 h. After that, free PPa and CPG/PPa NPs (200 ng/mL) were utilized to incubate these cells for 4 h, respectively. Subsequently, DCFH-DA (10 μg/mL) was added to 12-well cell-culture plates and further incubated for 30 min. These wells were irradiated using a 660 nm laser (30 mW/cm^2^, 10 min). Untreated laser group as a control. The ROS generation in 4T1 cells were determined by utilizing an inverted microscope.

Additionally, we further analyzed intracellular ROS generation quantitatively. 4T1 cells (1 × 10^4^ cells/well) were cultured in black 96-well cell-culture plates overnight. Subsequently, the process was the same as described above. Finally, microplate reader was used to detect the generation of cellular ROS (Thermo Scientific, USA).

### Cell viability evaluation

MTT assay was applied to determine the cytotoxicity of free PPa (78.125-1250 ng/mL), free CPG (93.75-1500 ng/mL), CPG/PPa mixture and CPG/PPa NPs (at an equivalent does of free PPa and CPG) against 4T1, RM-1, HepG2 and L02 cells. These cells (2000 cells/well) were cultured in 96-well plates for 12 h attachment, and then different concentrations of above formulations were added to these cells for 48 h. In terms of photodynamic cytotoxicity, cells were cultured with above drugs for 4 h, then these cells were irradiated with laser (660 nm, 30 mW/cm^2^) for 2 min. Afterwards, these cells were cultured another 44 h before MTT determination. Finally, the absorbency of the samples in 96-well plates was detected in 570 nm by a microplate reader. Furthermore, we also investigated the viability of 4T1, HepG2, RM-1 and L02 cells, which were incubated with high concentration of CPG (781.25-12500 ng/mL) according to the procedure mentioned above.

### Synergistic effect of CPG/PPa NPs in cells incubated with GSH-OEt

The synergistic effect CPG and PPa of CPG/PPa NPs was further investigated in 4T1, HepG2, and RM-1 cells incubated with GSH-OEt. Briefly, 4T1, HepG2, and RM-1 cells were seeded in 96-well culture plates at a density of 3000 cells/well and incubated 12 h. Then, these cells were preincubated with GSH-OEt (10 mM) for 2 h 38,39. Next, these cells were washed with PBS three times, free PPa (78.125-1250 ng/mL), free CPG (93.75-1500 ng/mL), CPG/PPa mixture and CPG/PPa NPs (at an equivalent does of free PPa and CPG) were added to these cells. After incubation 4 h, these cells were irradiated with laser (660 nm, 30 mW/cm^2^) for 2 min. After incubation another 44 h, the cell viability was assessed by standard MTT method. And combinational index (CI, 50% inhibition) was calculated by using CompuSyn software.

### Animal studies

The Sprague-Dawley rats used in the pharmacokinetic experiment and the Balb/c mice used in the anti-tumor studies conform to the Animal Ethics Committee of Shenyang Pharmaceutical University.

### Pharmacokinetic studies

Sprague-Dawley rats (200-220g) were employed to investigate *in vivo* pharmacokinetic behavior of CPG/PPa NPs. Rats were randomly divided into three groups (n=3). CPG/PPa mixture, non-PEGylated CPG/PPa NPs and CPG/PPa NPs (equivalent dose with 8 mg/kg of PPa) were intravenously injected into rats. At predesigned timepoints, about 500 μL blood samples was harvested from each the rat's ophthalmic vein. Then the plasma was obtained via centrifugation (1.3 × 10^4^ rpm, 10 min). Finally, the multifunctional microplate reader was employed to detect the concentration of PPa in the plasma.

### Biodistribution

4T1 tumor-bearing mice model was employed to investigate the biodistribution of CPG/PPa NPs. Briefly, the mice were first anesthetized utilizing isoflurane, 100 μL PBS containing 5 × 10^6^ 4T1 cells were implanted into the flank region of right back of female BABL/c mice. 200 uL PBS, free PPa solution (6mg/kg), CPG/PPa mixture and CPG/PPa NPs (at an equivalent does of PPa) were administrated intravenously via tail vein into the mice when the average volume of tumors reached around 400 mm^3^. At post 4 h, 12 h, 1 d and 3 d administration, the mice were killed. Afterwards, the major organs of each group (heart, liver, spleen, lung, kidney) and tumors were isolated. Finally, the fluorescence imaging and fluorescence intensity of major organs and tumors were analyzed by an *in vivo* imaging system (IVIS) (n=3). In addition, the biodistribution of non-PEGylated CPG/PPa NPs and CPG/PPa NPs at post 1 d administration was used to investigate the tumor penetration and tumor targeting of PEGylated nanoparticles.

### *In vivo* synergistic anti-tumor effect

4T1 breast tumor xenograft model was utilized to investigate anti-tumor effect of CPG/PPa NPs *in vivo*. The establishment of tumor model was the same as “Biodistribution”. After approximately 1 week, the tumor volume reached an average volume of around 200 mm^3^, the mice with tumors were divided into five groups randomly (n=5): control (saline), free CPG, PPa/L (free PPa + laser), CPG/PPa/L (CPG/PPa mixture + laser), CPG/PPa NPs/L (CPG/PPa NPs + laser). CPG/PPa physical mixture was prepared by saline containing 5% Cremophor RH 40 and 5% ethanol (v/v). Briefly, appropriate ethanol was added to the solid mixture of CPG and PPa (molar ratio of 2:1) and then Cremophor RH 40 was added to obtain CPG/PPa mixed solution, finally, diluted to the desired concentration with saline. PPa solution was prepared in the same way of CPG/PPa mixture. CPG/PPa NPs needed to be concentrated before administration. Ultrafiltration centrifugation was used to concentrate CPG/PPa NPs. The details were as follows: 2 mL CPG/PPa NPs were added into 4 mL ultrafiltration centrifuge tube (10KDa), and then concentrated to 600 µg/mL at 2500 rpm for 15 min. These formulations (6 mg/kg PPa, 7.2 mg/kg CPG) were intravenously administrated at intervals of 2 days (day 0, 2, 4, 6), and the laser treatment group of mice were irradiated with laser (660 nm, 200 mW/cm^2^) for 5 min 1. The body weight and tumor volume of mice were recorded every day. The calculation formula of tumor volume was as follows: V (mm^3^) = 1/2 (a × b^2^) (a: length, b: width). The mice were killed on day 11; then the blood of each mice was collected to evaluate the liver and renal function of each group of mice. The lung tissues were excised and stained with picric acid for imaging. And lung slices were collected from all mice groups to investigate the lung metastasis of different formulations. Then other major organs and tumors of each mice were also isolated and weighed. The hematoxylin and eosin (H&E) staining was used to estimate the pathological variations of the mice. In addition, coagulation indicators including prothrombin time (PT) and activated partial thromboplastin time (APTT) of different treated groups were used to evaluate the safety of CPG/PPa NPs.

### Statistical analysis

All data were calculated and expressed as mean ± SD. Two tailed t-test or one-way analysis of variance (ANOVA) were used to analyze the differences between comparative groups. Significant statistical differences were evaluated at *P* < 0.05.

## Results and Discussion

### Preparation and characterization of non-PEGylated CPG/PPa NPs

 We prepared the non-PEGylated CPG/PPa NPs by one-step nano-precipitation technique. As showed in Figure S1A, the image of TEM displayed that non-PEGylated CPG/PPa NPs had uniform spherical nanostructures. The dynamic light scattering (DLS) exhibited that the average size and zeta potential of non-PEGylated were approximately 97 nm (Figure S1B) and about -23 mV (Figure S1C), respectively. In Figure S1D, the size of non-PEGylated CPG/PPa NPs increased and some larger particles appeared after incubation with PBS containing 10% FBS for 4 h, indicating that nanoparticles were unstable.

Computational simulations based on detailed classical and/or quantum analysis have been employed to study the drug-drug interaction at the molecular level 37. Especially molecular dynamics (MD) simulations, could help to predict the assembly mechanism of nanoparticles 40. Therefore, the computational simulations and experimental validation were collectively investigated to co-assembling mechanism of CPG and PPa. As illustrated in Figure S2A, MD simulations revealed that hydrophobic forces existed between the porphyrin ring of PPa and hydrophobic chain of CPG, and π-π stacking existed between the planar conjugated aromatic rings of CPG and PPa. In Figure S2B, evident red shift and widened absorption peak were observed in the UV absorbance spectrum of non-PEGylated CPG/PPa NPs compared with free PPa. Additionally, following the addition of SDS (0.2% w/v), the UV adsorption value of non-PEGylated CPG/PPa NPs was decreased evidently. These results implied that the π-π stacking and strong hydrophobic forces were involved in the co-assembly process. Moreover, the infrared spectra of CPG, PPa, CPG/PPa physical mixture, and non-PEGylated CPG/PPa NPs were characterized. The peak intensity of carbonyl (1727.0 cm^-1^) in carboxyl group of PPa in NPs was weaker, compared to CPG/PPa mixture and PPa. In addition, the broadening and shift to lower wavenumbers of the hydroxyl (-OH) peak of ester bond in CPG were observed (Figure S2C). The results suggested that CPG and PPa could form intermolecular hydrogen bond depending on carbonyl of carboxyl group (PPa) and hydroxyl group of ester bond (CPG). Moreover, some chemical materials including NaCl, SDS, and urea were used to treat non-PEGylated CPG/PPa NPs. NaCl was used to shield the electrostatic action between CPG and PPa in non-PEGylated CPG/PPa NPs, SDS to disaggregate the hydrophobic action between CPG and PPa in NPs, urea to destroy the hydrogen bonds between CPG and PPa in NPs. The change in particle size of non-PEGylated CPG/PPa NPs was illustrated in Figure S2D, the effective disaggregation of non-PEGylated CPG/PPa NPs was observed by adding NaCl, SDS and urea 41,42. The results of experiment and computational simulations collectively validated that co-nanoassemblies were formed by hydrophobic interactions, hydrogen bond, π-π stacking and electrostatic interactions between CPG and PPa.

### Preparation and characterization of CPG/PPa NPs

We firstly studied the cytotoxic effect of the different molar ratios (5:1, 2:1, 1:1, 1:2 and 1:5) of CPG to PPa by calculating the CI (50% inhibition) in 4T1 cells. Among these, CIs of (CPG:PPa) 5:1 and 2:1 were 0.86 and 0.57 in 4T1 cells, exhibiting the great synergistic effect (Table S1). The CPG/PPa NPs could be prepared using a one-step precipitation method. Then, we also investigated the mean diameter and polydispersion index (PDI) of various molar ratios CPG/PPa NPs. The mean diameter and PDI of 2:1 (CPG:PPa) CPG/PPa NPs were smaller than that of 5:1 (CPG:PPa) CPG/PPa NPs (Table S1). So, CPG/PPa NPs with the molar ratio of 2:1 (CPG:PPa) were used for the further studies.

**Figure 2:**
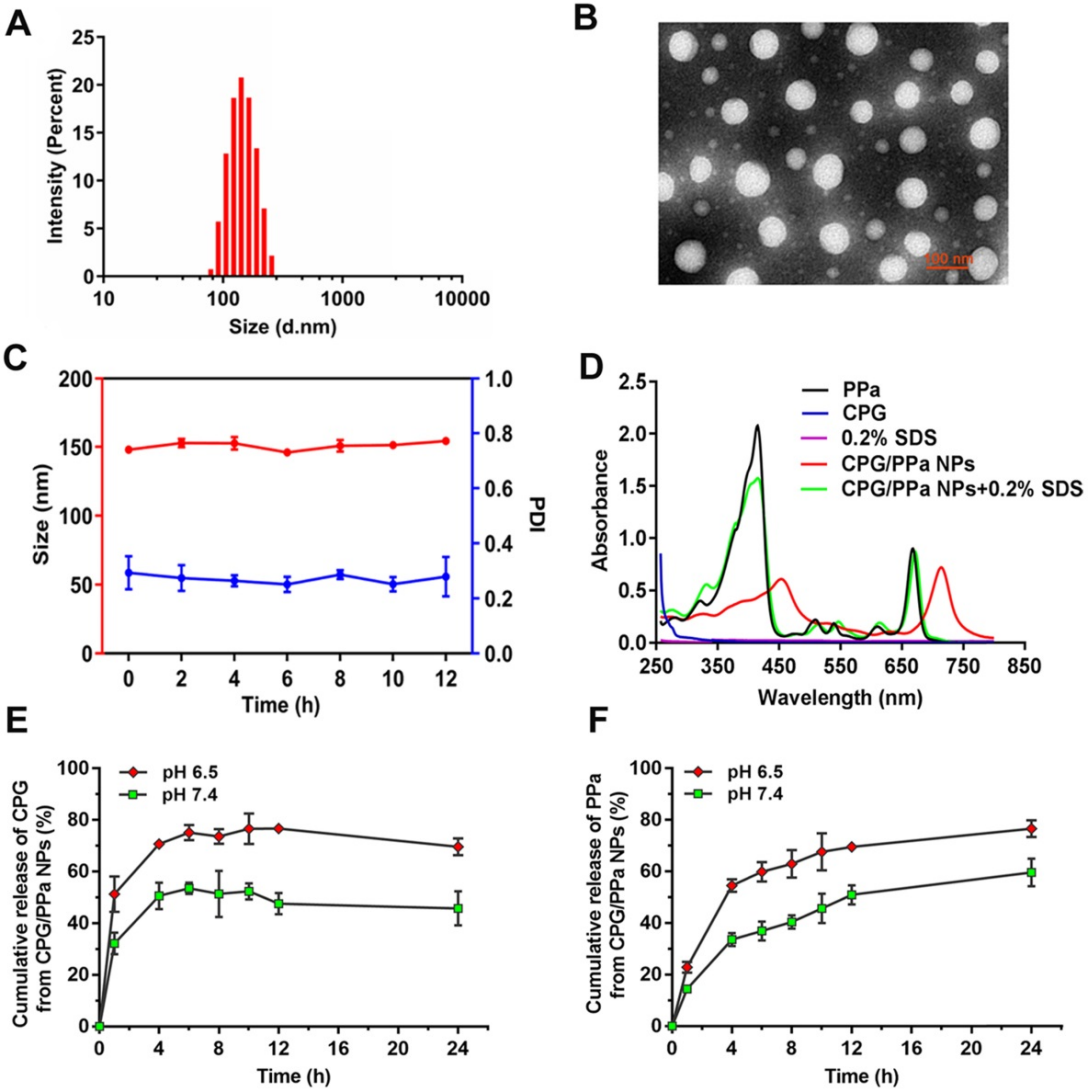
** Characterization of co-assembled nanoparticles *in vitro*. (A)** Intensity distribution profile of Size, **(B)** TEM image of CPG/PPa NPs, **(C)** Colloidal stability of CPG/PPa NPs (n=3), **(D)** UV absorption spectra at 250-800 nm wavelength of 0.2% SDS, CPG/PPa NPs containing 0.2% SDS, PPa solution, CPG solution, and CPG/PPa NPs, **(E)** *In vitro* accumulative drug release curves of CPG from CPG/PPa NPs in PBS (pH 6.5, 7.4) within 24 h, **(F)** *In vitro* accumulative drug release curves of PPa from CPG/PPa NPs in PBS (pH 6.5, 7.4) within 24 h (n=3).

From the DLS and TEM analyses (Figure 2A, B), it could be observed that CPG/PPa NPs had uniform spherical nanostructures, with an average diameter of ~140 nm and zeta potential of ~-24 mV (Figure S3). As illustrated in Figure 2C and Figure S4, the size of CPG/PPa NPs were barely changed in PBS (pH 7.4), RPMI 1640 and DMEM medium supplemented with 10% FBS in shaking table (37 °C), indicating the good colloidal stability. Moreover, the size of CPG/PPa NPs was barely changed in plasma with 12 h, demonstrating that NPs had good colloidal stability in plasma (Figure S5).

The encapsulation efficacy of CPG and PPa were approximately 81.6% and 76.5%, respectively, as measured by HPLC and microplate reader. The loading efficiency of CPG and PPa were about 37.1% and 29.0%, respectively.

Computational simulations (Figure S2A) and IR spectra (Figure S2C) demonstrated that CPG and PPa could be assembled into nanoparticles depending on π-π stacking, hydrophobic forces and hydrogen bond. As presented in Figure 2D, evident red shift and widened absorption peak were observed in the UV absorbance spectra of CPG/PPa NPs compared with free PPa. Additionally, the UV adsorption value of CPG/PPa NPs was decreased obviously with the addition of SDS (0.2% w/v), and the UV/vis curve of CPG/PPa NPs was back to the original position of the PPa. Moreover, the size of CPG/PPa NPs increased significantly after incubation with different concentrations of NaCl, SDS and urea (Figure S6A). As depicted in Figure S6B, the images of TEM showed that the size of CPG/PPa NPs increased with the addition of NaCl, SDS, and urea, indicating the effective disaggregation of CPG/PPa NPs in the above chemical materials. These results implied that the π-π stacking, hydrogen bond, hydrophobic and electrostatic interactions were involved in the co-assembly process of CPG/PPa NPs.

As depicted in Figure S7, the position of absorption peak of fluorescence spectrum of CPG/PPa NPs barely changed compared to free PPa. However, the fluorescence intensity of CPG/PPa NPs decreased compared to free PPa, which might be due to exciton migration during molecules stacking and the changed molecular conformation involved in co-assembly 43,44.

*In vitro* release of CPG/PPa NPs, the release rates of CPG and PPa were faster in acidic medium (pH 6.5) compared with the neutral medium (pH 7.4) (Figure 2E-F). Approximately, 50% CPG and PPa were released from CPG/PPa NPs within 24 h in the neutral medium (pH 7.4), while >70 % CPG and PPa were released in the acidic medium (pH 6.5). This indicated that CPG could be released rapidly from CPG/PPa NPs under the acidic conditions. As shown in Figure S8A, the size of CPG/PPa NPs increased significantly at pH 6.5, but almost no change at pH 7.4. Moreover, after incubation CPG/PPa NPs in PBS (pH 6.5) for 2 h, the particle size of CPG/PPa NPs increased obviously, and the change of particle size was time-dependent, indicating that NPs were unstable in pH 6.5 PBS (Figure S8B). The results indicated that the expanded structure of NPs in PBS (pH 6.5) might be due to protonation of the tertiary group of CPG molecule in an acidic environment and weakened the interactions between CPG and PPa, leading to a pH-responsive drug release behavior of the CPG/PPa NPs 45,46. The pH-responsive drug release profiles would be beneficial to their stable nanostructures in the blood circulation without leakage and then rapid release followed by cellular endocytosis in tumor cells.

### The content and activity of CYP2C19 and relative content of GSH detection in cells

**Figure 3:**
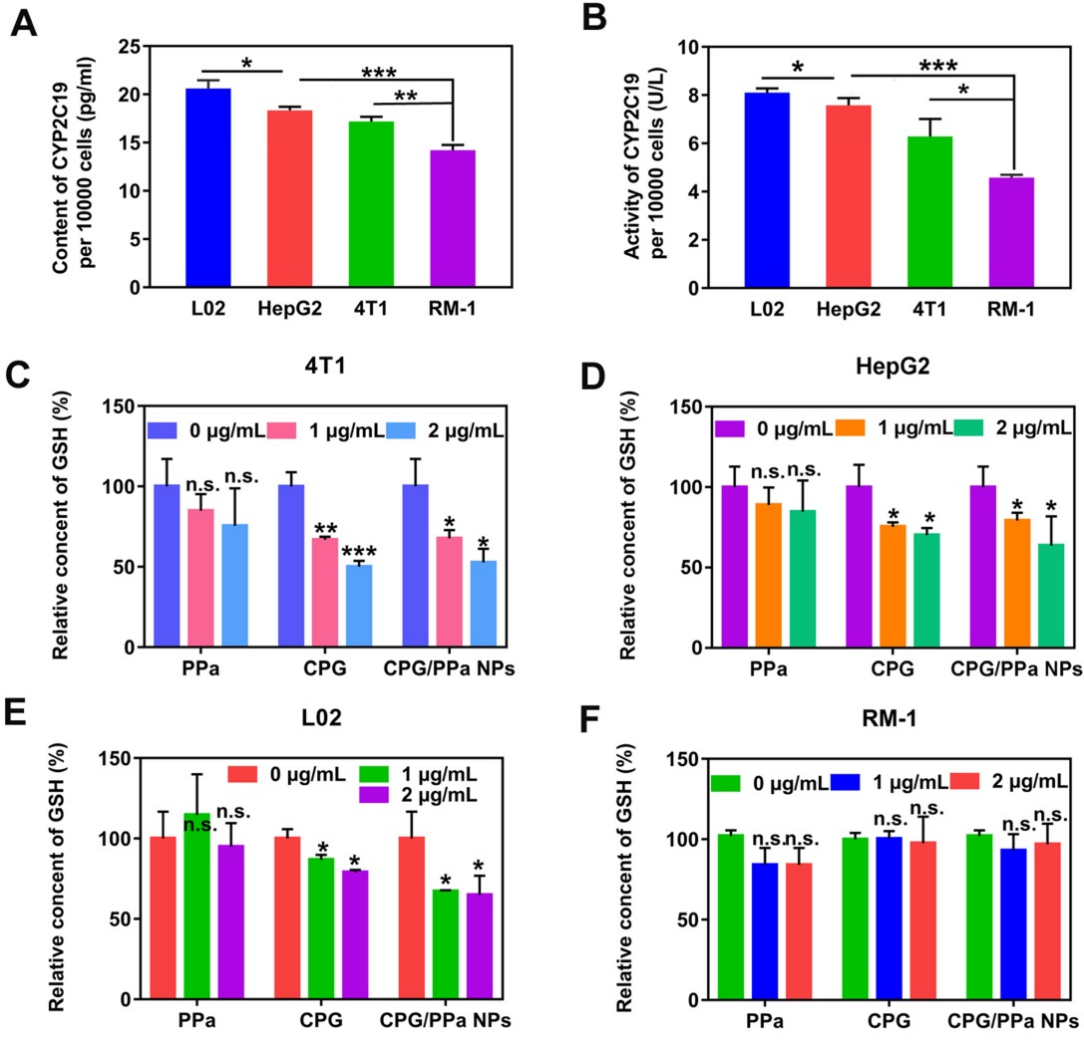
** Intracellular GSH level and CYP2C19 content and activity of 4T1, RM-1, HepG2 and L02 cells. (A)** CYP2C19 content of 4T1, RM-1, HepG2 and L02 cells, **(B)** CYP2C19 activity of 4T1, RM-1, HepG2 and L02 cells. **(C)** GSH relative level of 4T1 cells, **(D)** GSH relative level of HepG2 cells, **(E)** GSH relative level of L02 cells, **(F)** GSH relative level of RM-1 cells, incubated with various concentrations (0 ug/mL, 1 ug/mL, 2 ug/mL) of CPG solution, PPa solution and CPG/PPa NPs (n=3, n.s. no significance, *P < 0.05, **P < 0.01, ***P < 0.001).

Inspired by the metabolic mechanism of CPG (Figure S9), we investigated the content and activity of CYP2C19 in different tumor cells (HepG2, 4T1, RM-1 cells) and a normal cell (L02 cells). As presented in Figure 3A and 3B, significantly higher content and activity of CYP2C19 were measured in L02 cells than those in the other three tumor cells. The content and activity of CYP2C19 followed the order of L02 cells > HepG2 cells > 4T1 cells > RM-1 cells.

High content and activity of CYP2C19 in HepG2 and 4T1 tumors would better metabolize CPG to consume intracellular GSH. Thus, we further assessed the variation of GSH content in the four cells when incubated with different concentrations of CPG, PPa and CPG/PPa NPs. As shown in Figure 3C-E, the intracellular GSH level could be significantly decreased in 4T1, HepG2 and L02 cells incubated with different concentrations of CPG, and CPG/PPa NPs. The decrease degree of GSH level would be in proportion to the CPG or CPG/PPa NPs concentration. Nevertheless, no change of intracellular GSH level was found in RM-1 cells, probably due to lower activity of CYP2C19 in RM-1 cells (Figure 3F). Additionally, the GSH level in four cells was no obviously change with free PPa treatment (Figure 3C-F). It was further suggested that released CPG from CPG/PPa NPs could consume intracellular GSH, but not for PPa. To further confirm that the active metabolites of CPG could conjugate with GSH, 4T1 cells were treated with CPG (1 μg/mL) for 48 h. Then the highly precise molecular weight analysis was performed to confirm the conjugate. The theory value of the molecular weight of CPG-SS-GSH conjugate were 660.1327 and 662.1297 due to the existence of Cl isotopes. The results of mass spectra demonstrated that the molecular weight of the conjugate of GSH with CPG active metabolites was 661.29693 [M + H]^+^ and 663.53568 [M + H]^+^, proving the formation of CPG-SS-GSH conjugate (Figure S10).

### Cellular uptake and ROS detection

**Figure 4:**
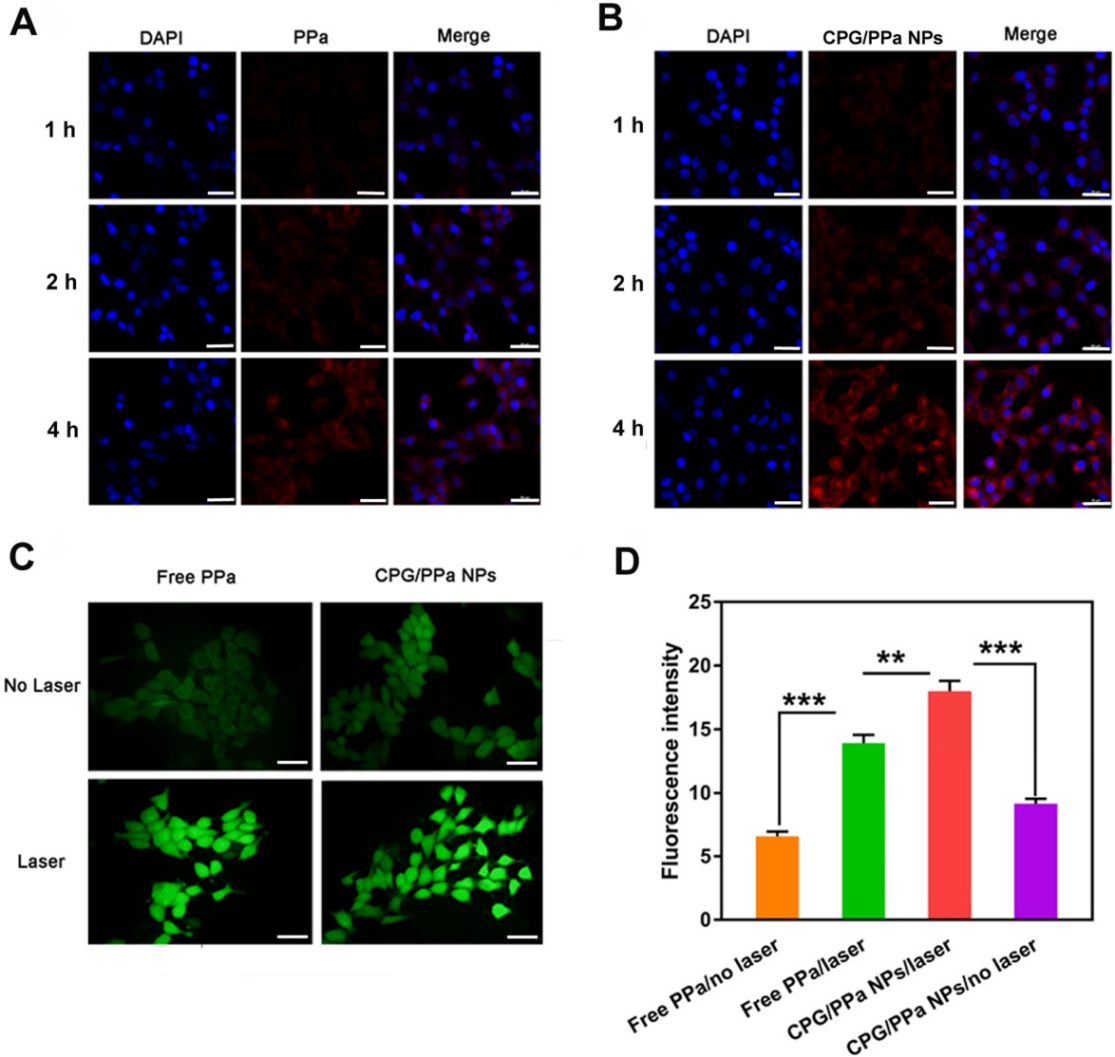
**Intracellular uptake and ROS generation of co-nanoassemblies. (A)** Confocal imaging of 4T1 intracellular uptake of free PPa at 1 h, 2 h and 4 h, **(B)** Confocal imaging of intracellular uptake of CPG/PPa NPs at 1 h, 2 h and 4 h (the scale in the figure was 20 µm). **(C)** Intracellular ROS generation of free PPa and CPG/PPa NPs in 4T1 cells with/without laser irradiation (30 mW/cm^2^, 10min) (the scale represents 20 μm). **(D)** The fluorescence intensity of DCF in 4T1 cells incubated with free PPa and CPG/PPa NPs with/without laser irradiation (30 mW/cm^2^, 10min) (n=3, **P < 0.01, ***P < 0.01).

The internalization and cellular uptake of CPG/PPa NPs in 4T1 cells at different time intervals were explored by CLSM. 4T1 cells were incubated with CPG/PPa NPs and free PPa for 1 h, 2 h and 4 h, respectively. As depicted in Figure 4A and 4B, PPa presented red fluorescence in cytoplasm. Compared with free PPa solution, CPG/PPa NPs exhibited much stronger fluorescent signal in 4T1 cells. The results suggested that the CPG/PPa NPs had higher cellular uptake efficiency than free PPa. In addition, the intracellular uptake profile showed a time-dependent increase manner during incubation of CPG/PPa NPs.

Furthermore, HPLC was used for quantitative determination of the cellular uptake of CPG/PPa NPs or free PPa. As illustrated in Figure S11, compared with free PPa, CPG/PPa NPs had higher cellular uptake efficiency at both 2 h and 4 h. The results were in a good accordance with the analytical results of CLSM.

DCFH-DA staining was utilized to investigate 4T1 intracellular ROS generation. Followed by incubation of CPG/PPa NPs and free PPa in 4T1 cells for 4 h, these cells were treated with or without the laser irradiation (660 nm, 30 mW/cm^2^, 10 min). As depicted in Figure 4C, the groups with the laser irradiation exhibited stronger intracellular fluorescence intensity for CPG/PPa NPs and free PPa groups than those without the laser irradiation exposure. More importantly, CPG/PPa NPs generated much higher ROS level than free PPa, further confirming the GSH-depleting capability of CPG active metabolites. The quantitative determination of the generated ROS in 4T1 cells with/without the laser irradiation showed the similar results (Figure 4D).

### *In vitro* cytotoxicity evaluation and synergistic effect of CPG/PPa NPs in cells incubated with GSH-OEt

**Figure 5:**
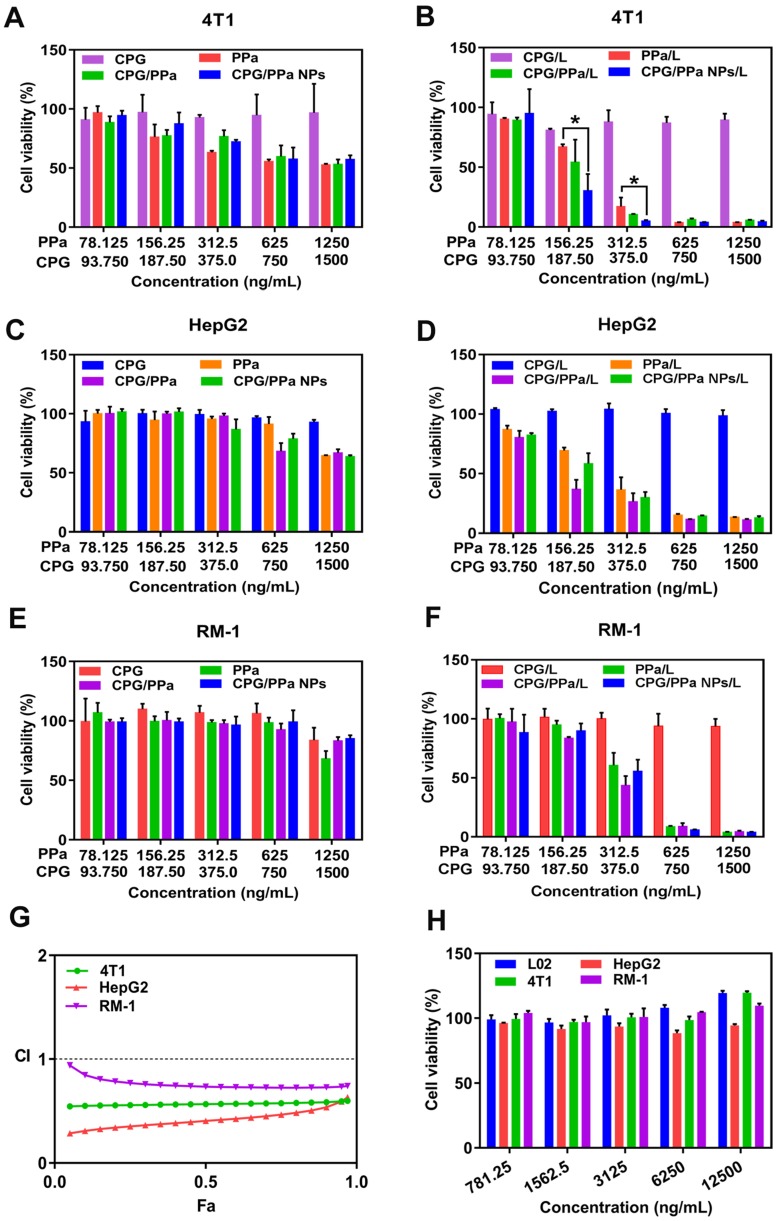
***In vitro* cell viability of 48 h incubated with different formulations against 4T1, HepG2, RM-1 and L02 cells. (A), (C)** and **(E)** The viability of 4T1, HepG2 and RM-1 cells, which were incubated with free PPa solution, CPG/PPa mixture, free CPG solution and CPG/PPa NPs without laser. **(B), (D)** and **(F)** The viability of 4T1, HepG2 and RM-1 cells, which were incubated with free PPa solution, CPG/PPa mixture, free CPG solution and CPG/PPa NPs under laser. **(G)** Combination index of CPG/PPa NPs in 4T1, HepG2 and RM-1 cells. **(H)** Cytotoxicity of free CPG in 4T1, HepG2, RM-1 and L02 cells (n = 3, *P < 0.05). (Figure 5A-F, 78.125, 156.25….1250 ng/mL in the horizontal ordinate represented the concentration of PPa, and 93.750, 187.50….1500 ng/mL in the horizontal ordinate represented the concentration of CPG).

The cytotoxicities of CPG/PPa NPs, CPG/PPa physical mixture, free PPa and free CPG in 4T1, RM-1, HepG2 and L02 cells were estimated by MTT determination. As illustrated in Figure 5A-F, CPG/PPa NPs, CPG/PPa mixture and free PPa showed the notably enhanced cytotoxicity towards the three tumor cells under laser irradiation (660 nm, 30 mW/cm^2^, 2 min), compared to those without laser irradiation. In addition, both CPG/PPa NPs and CPG/PPa mixture exhibited higher cytotoxicity than free PPa under laser irradiation. Free CPG exhibited no obvious cytotoxicity in three tumor cells (4T1, RM-1 and HepG2) whether laser irradiation or not. As illustrated in Figure S12, CPG/PPa NPs, CPG/PPa physical mixture, free PPa showed cytotoxic effect on L02 cells under laser irradiation. However, the above formulations and free CPG exhibited no significant cytotoxicity against on L02 cells without laser irradiation. Since only the tumor sites of mice were exposed to laser irradiation after administration of CPG/PPa NPs, indicating that above formulations were safe to the normal cells. In Figure 5G, the CI of CPG/PPa NPs was less than 1, indicative of the synergistic cytotoxic effect of CPG and PPa in three tumor cells. However, the synergistic effect of CPG/PPa NPs in RM-1 cells was worse than those in 4T1 and HepG2 cells, due to the low activity of CYP2C19 in RM-1 cells. The results were also consistent well with the GSH level in the three tumor cells following the addition of CPG. Moreover, free CPG showed no noticeable cytotoxicity in three tumor cells (4T1, RM-1, HepG2 cells) and a normal cell line (L02 cells) at concentration range from 781.5 to 12500 ng/mL (Figure 5H).

In order to better prove that the synergistic effect between clopidogrel and PPa was achieved by the reduction of GSH. The synergistic effect of CPG/PPa NPs was assessed by these cells incubated with GSH-OEt. As depicted in Figure S13. CPG/PPa NPs exhibited strong cytotoxic to 4T1, HepG2, RM-1 cells treated with GSH-OEt, and showed good synergistic effect (CI<1). The results revealed that synergistic effect of CPG/PPa NPs had barely changed in these cells incubated with GSH-OEt.

### Pharmacokinetic studies

**Figure 6:**
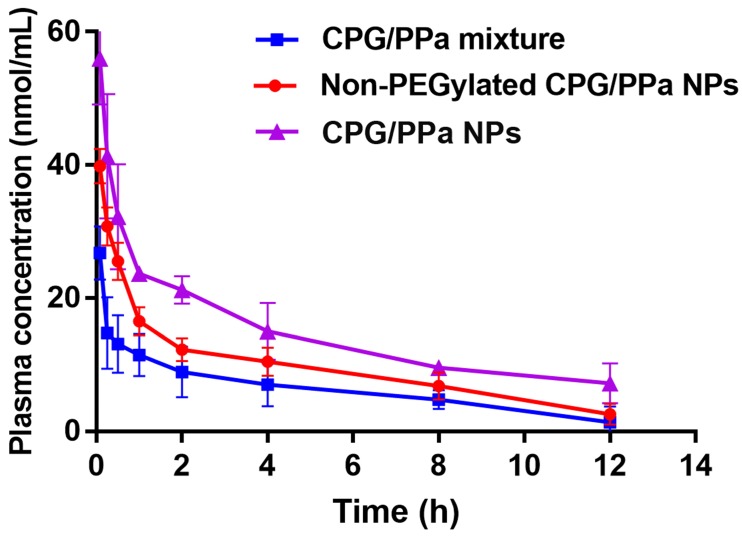
The curves of PPa concentration from CPG/PPa mixture, non-PEGylated CPG/PPa NPs and CPG/PPa NPs with time after a single administration via tail vein at a PPa equivalent of 8.0 mg/kg (n=3).

In the assembled nano-systems, DSPE-PEG_2k_ was used to prolong circulation time CPG/PPa NPs in blood. Therefore, we evaluated the *in vivo* pharmacokinetic characteristics of CPG/PPa mixture, non-PEGylated CPG/PPa NPs and CPG/PPa NPs. The molar concentration-time curves and the main pharmacokinetic parameters were illustrated in Figure 6 and Table S2, respectively. As depicted in Figure 6, CPG/PPa NPs showed that the increased area under curve (AUC_0-12h_) was responsible for the prolonged blood circulation time, compared with CPG/PPa mixture and non-PEGylated CPG/PPa NPs. The results suggested that PEGylation could enhance the circulation time of CPG/PPa NPs *in vivo*.

### *In vivo* biodistribution

**Figure 7:**
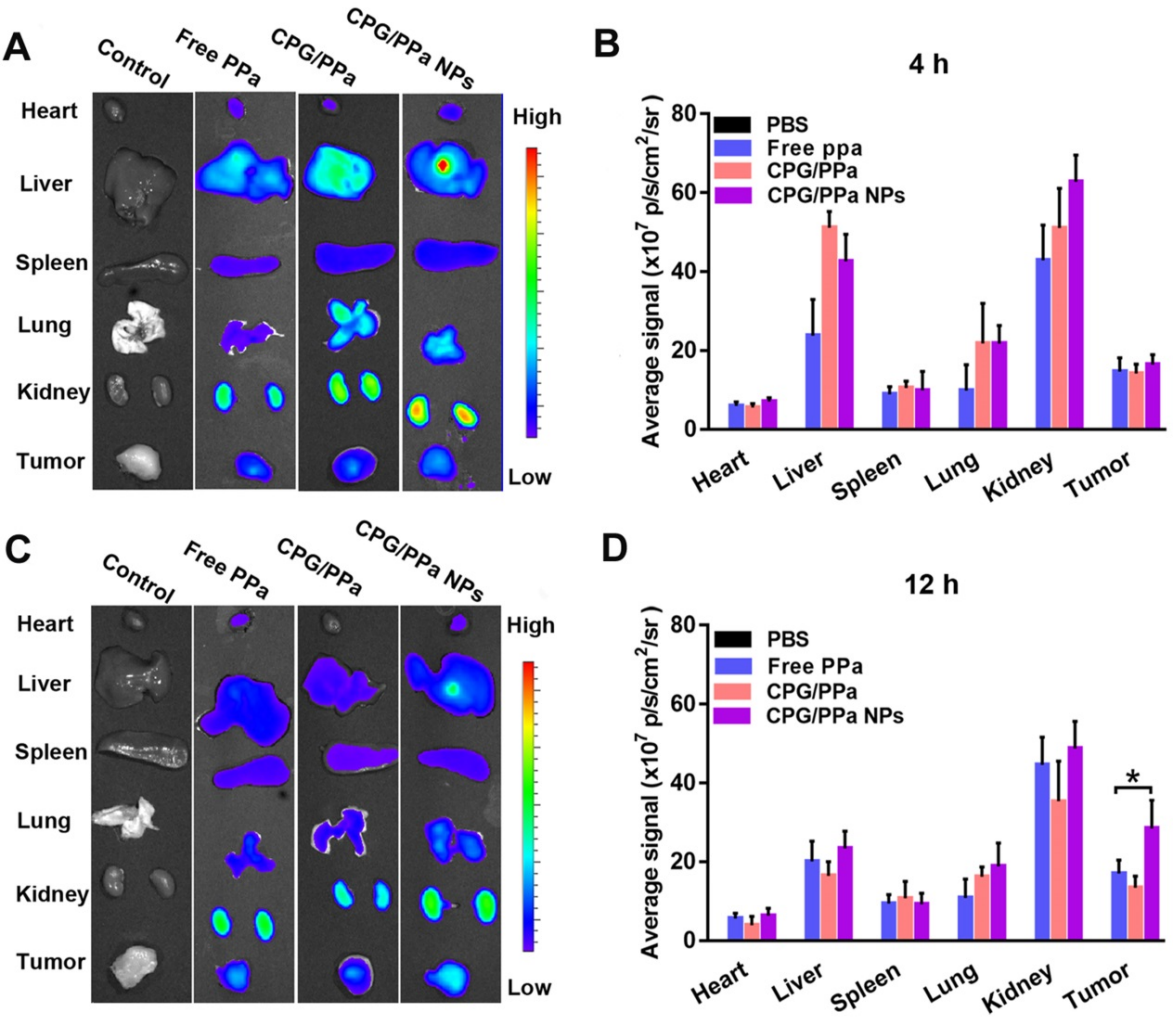
** Biodistribution of PBS, free PPa, CPG/PPa and CPG/PPa NPs in 4T1 tumor-bearing BALB/c mice. (A)** *In vitro* fluorescence imaging of major organs and tumors at 4 h, **(B)** Quantitative analysis average fluorescence intensity at 4 h; **(C)** *In vitro* fluorescence imaging of major organs and tumors at 12 h; **(D)** Quantitative analysis average fluorescence intensity at 12 h (n = 3, *P < 0.05).

To determine the accumulation of CPG/PPa NPs in tumors and the optimal illumination time for *in vivo* anti-tumor study, the test of biodistribution was carried out in 4T1 tumor-bearing mice. As shown in Figure 7A, C, *in vivo* biodistribution of main organs of the CPG/PPa NPs was similar to free PPa and CPG/PPa mixture. Livers and kidneys displayed stronger fluorescence than other organs at 4 h post-administration. Strong fluorescence in liver and kidneys might be due to the phagocytosis of partial drugs through reticuloendothelial system of the liver and excretion by kidneys, respectively 47-49. However, in comparison with free PPa and CPG/PPa mixture, CPG/PPa NPs exhibited higher fluorescence intensity in tumor sites at 12 h post-administration, the fluorescent intensity in tumors increased over time from 4 to 12 h (Figure 7A-D). As showed in Figure S14, the accumulation of CPG/PPa NPs in tumor tissues was significantly higher than that of free PPa and CPG/PPa at 1 d and 3 d post administration. Additionally, the fluorescent signals in major organs and tumors were very weak at 3 d post administration, indicating that these formulations were mostly eliminated. Since the CPG/PPa NPs were PEGylated, PEG could increase the circulation time of nanoparticles (Figure 6), and enhanced permeability and retention (EPR) effects could enhance the accumulation of nanoparticles in tumor sites 50. CPG/PPa NPs exhibited higher accumulation in tumor tissues than that of non-PEGylated CPG/PPa NPs at 1 d post administration (Figure S15), which was attributed to the extended blood circulation time of PEGylated nanoparticles. The results revealed that the enhanced accumulation of CPG/PPa NPs in tumor sites was ascribed to extended blood-circulation time and EPR effects.

### *In vivo* synergistic anti-tumor effect

**Figure 8:**
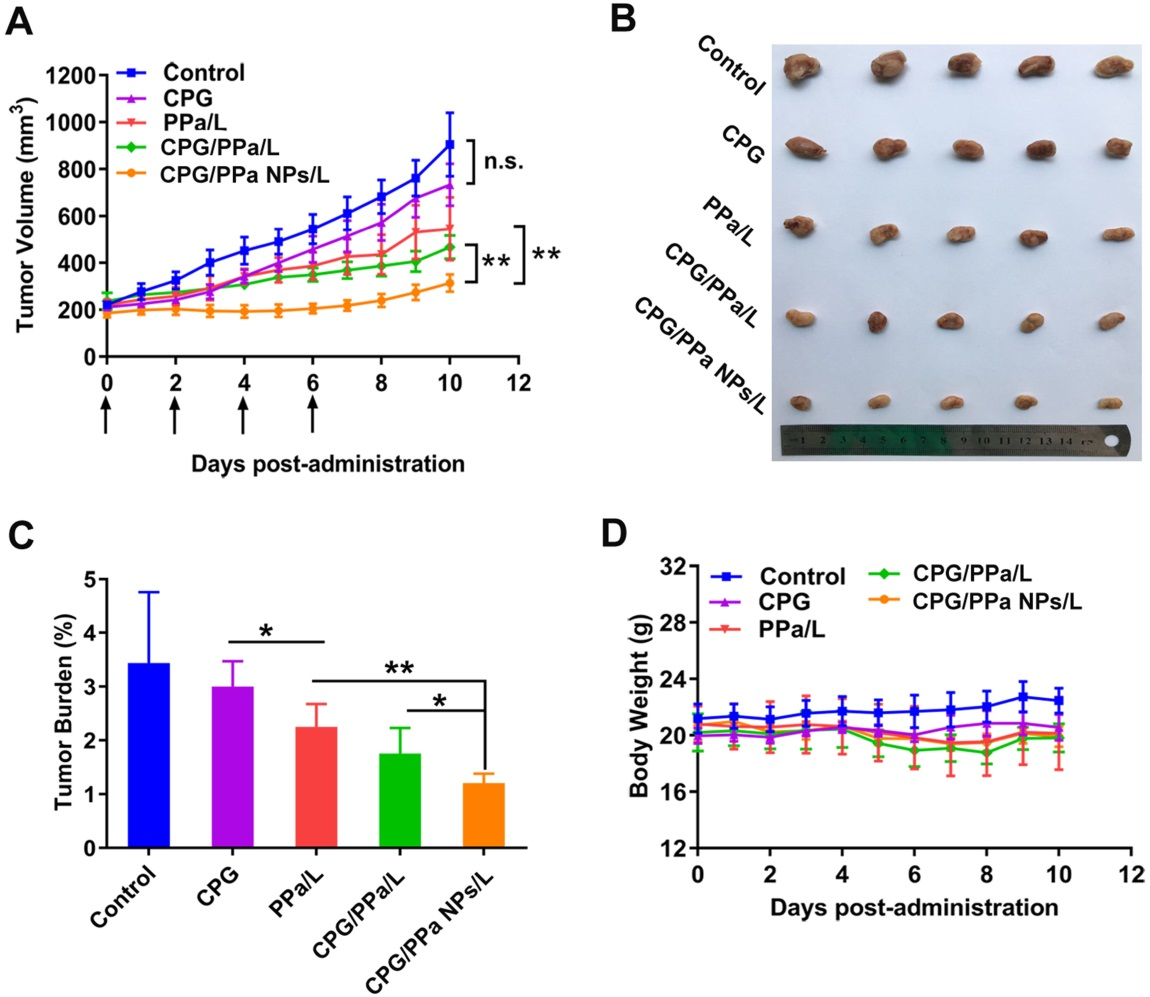
** CPG/PPa NPs exhibited synergistic anti-tumor effect in 4T1 tumor-bearing BALB/c mice with 660 nm laser irradiation (200 mW/cm^2^, 5 min). (A)** Tumor growth curves, which treated with saline, CPG, PPa, CPG/PPa, and CPG/PPa NPs (administration via tail vein on day 0, 2, 4, 6). **(B)** Images of excised tumors on day 11. **(C)** The tumor burden of mice after different treatments. **(D)** Average body weight of mice in different administration groups on day 0-11. (n=5, n.s. no significance, *P < 0.05, **P < 0.01).

The *in vivo* anti-tumor effect of CPG/PPa NPs was investigated in the 4T1 bearing-tumor mice. GSH level was higher in 4T1 cells compared with the other two tumor cells (RM-1 and HepG2) (Figure S16). Furthermore, the activity and content of CYP2C19 in 4T1 cells were also obviously higher than that in RM-1 cells (Figure 3A-B). Therefore, based on the above factors, 4T1 tumors were selected a tumor model. The tumor size and weight of each mice were determined daily. As shown in Figure 8A-C, the tumor volume treated with saline, reached approximately 1000 mm^3^ at day 11, and CPG groups also barely inhibited tumor growth. PPa/L and CPG/PPa/L group showed the moderate anti-tumor activity compared with the control group. Remarkably, CPG/PPa NPs/L group exhibited the best tumor growth inhibition effect, which was attributed to the following reasons: (i) long circulation time of CPG/PPa NPs *in vivo* and high accumulation in tumors. (ii) cooperative ROS-enhancing effect of CPG and PPa under laser irradiation.

Considering the important roles of platelet in the metastatic cascade, the influence of CPG/PPa NPs on tumor metastasis was further investigated. As shown in Figure S17, tumor metastasis was more effectively inhibited by CPG/PPa NPs when compared with other groups. The anti-metastasis activity of CPG/PPa NPs should be attributed to the inhibition of platelet aggregation by CPG 51,52.

The biosafety and biocompatibility of all the studied groups were further evaluated. As shown in Figure 8D, average body weight of mice in different administration groups barely changed on day 0-11. Besides, in terms of hematological results, no significant difference among the five groups was observed, indicating that there was no significant hepatic and renal toxicity (Figure S18). H&E staining results also showed no obvious histological variation in the main organs of mice in all the groups (Figure S19). Moreover, there was no significant difference in the five treated groups in terms of PT and APTT, suggesting that CPG/PPa NPs showed no significant effect on these coagulation indicators (Figure S20). All these results indicated that CPG/PPa NPs had good biosafety and biocompatibility *in vivo*.

As a classical anti-platelet prodrug, the metabolic mechanism of CPG was clear, and the biological safety was clearly proven 30. With the remote tumor-targeting laser radiation, CPG/PPa NPs should demonstrate the good systematic safety. In addition, CPG, PPa, CPG/PPa mixture and CPG/PPa NPs were no significant hepatic and renal toxicity in terms of hematological results (Figure S19).

## Conclusions

Inspired by depleting GSH capability via CYP2C19-activated CPG metabolite, we successfully fabricate a facile co-assemble CPG/PPa NPs for auto-enhanced antitumor photodynamic therapy. The co-assembly molecular mechanism between CPG and PPa is put forward based on the computational simulations and experimental validation. CPG/PPa NPs present higher cellular uptake efficiency, greater ROS-generated level and better accumulation in tumor tissues compared with free PPa, resulting in improved photodynamic cytotoxicity. On account of the synergistic effect of CPG and PPa, CPG/PPa NPs exhibit the best anti-tumor efficiency in 4T1 tumor-bearing mice *in vivo.* Our results pave the way for the rational design strategy of the combinational PDT-based cancer therapy. Notably, the CYP2C19 level in tumor cells exerts crucial influence on the synergistic antitumor effect of CPG/PPa NPs, due to its important role in the activation of CPG. Therefore, tumors types and the expression heterogeneity of CYP2C19 should be fully taken into consideration for the potential clinical translation of CPG/PPa NPs in the future.

## Supplementary Material

Supplementary figures and tables.

## Abbreviations

ROS: reactive oxygen species
PDT: photodynamic therapy
PSs: photosensitizers
GSH: glutathione
CPG: clopidogrel
PPa: pyropheophorbide a
NPs: nanoparticles
BSO: L-buthionine sulfoximine
ADP: adenosine diphosphate
DSPE-PEG_2k_: 1,2-distearoylsn-glycero-3-phosphoethanolamine-N-[methoxy(polyethyleneglycol)-2000
DCFH-DA: 2,7-Dichlorodihydrofluorescein diacetate
GSH-OEt: glutathione reduced ethyl ester
DAPI: 4',6-diamidino-2-phenylindole
MTT: 3-(4,5-dimethyl-2-thiazolyl)-2,5-diphenyl-2-H-tetrazolium bromide
FBS: fetal bovine serum
CI: combination index
UV: ultraviolet
TEM: transmission electron microscopy
EE: encapsulation efficiency
LE: loading efficiency
HPLC: high-performance liquid chromatography
CLSM: confocal laser scanning microscopy
H&E: hematoxylin and eosin
PT: prothrombin time
APTT: activated partial thromboplastin time
DLS: dynamic light scattering
MD: molecular dynamics
PDI: polydispersion index
EPR: enhanced permeability and retention
