# Supplementary material for: Cytochrome P450 enzyme-mediated auto-enhanced photodynamic cancer therapy of co-nanoassembly between clopidogrel and photosensitizer
Source: Theranostics. 2020 Apr 15;10(12):5550–64. doi: 10.7150/thno.42633 (PMC7196307; doi:10.7150/thno.42633)
Supplement: Supplementary file 1 — Supplementary figures and tables. [file thnov10p5550s1.pdf]

Supplementary Material

Cytochrome P450 enzyme-mediated auto-enhanced photodynamic cancer therapy of co-nanoassembly between clopidogrel and photosensitizer

Authors: Qiu Wang[a,1], Mengchi Sun[a,1], Dan Li[a], Chang Li[a], Cong Luo[a], Zhaomeng Wang[a], Wenjuan Zhang[a], Zimeng Yang[a], Yao Feng[b], Shuang Wang[c], Zhonggui He[a], Haotian Zhang[d], Qiming Kan[d], Wei Sun[e,*], Jin Sun[a,*]

Affiliations:

[1]Both authors contributed equally

[a]Wuya College of Innovation, Shenyang Pharmaceutical University, Shenyang, 110016, P. R. China

[b]Key Laboratory of Structure-Based Drug Design and Discovery, Ministry of Education, Shenyang Pharmaceutical University, Shenyang, 110016, P. R. China

[c]School of Pharmacy, Shenyang Pharmaceutical University, Shenyang, 110016, P. R. China

[d]School of Life Science and Biopharmaceutics, Shenyang Pharmaceutical University, Shenyang, 110016, P. R. China

[e]College of Medical Device, Shenyang Pharmaceutical University, Shenyang, 110016,

P. R. China

***Corresponding Author:**

Prof. Jin Sun Ph.D

Wuya College of Innovation, Shenyang Pharmaceutical University, Shenyang, Liaoning, 110016, P. R. China

Tel/Fax: +86-24-23986321

E-mail: sunjin@syphu.edu.cn

Dr. Wei Sun

College of Medical Device, Shenyang Pharmaceutical University, Shenyang, Liaoning, 110016, P. R. China

Tel：+86-24-43520356

E-mail: sunwei19801208@163.com

Table S1. Optimization of molar ratios of CPG to PPa

Molar ratios of CPG to PPa	Z-Average (d. nm)	PDI	CI (50% inhibition)
1:5	120.3 ± 6.704	0.240 ± 0.032	3.1
1:2	126.3 ± 3.915	0.257 ± 0.057	3.7
1:1	91.17 ± 4.682	0.268 ± 0.039	3.8
2:1	141.9 ± 4.884	0.119 ± 0.069	0.57
5:1	268.3 ± 8.130	0.165 ± 0.144	0.86

Table S2. Pharmacokinetic parameters of PPa from CPG/PPa mixture, non-PEGylated CPG/PPa NPs and CPG/PPa NPs (n=3).

Formulations	AUC_{0-12} (nmol h/mL)	$t_{1/2}$ (h)
CPG/PPa mixture	87.408 ± 4.360	4.93 ± 0.442
non-PEGylated CPG/PPa NPs	117.2 ± 15.055	4.813 ± 0.704
CPG/PPa NPs	177.561 ± 9.648	5.9 ± 1.202

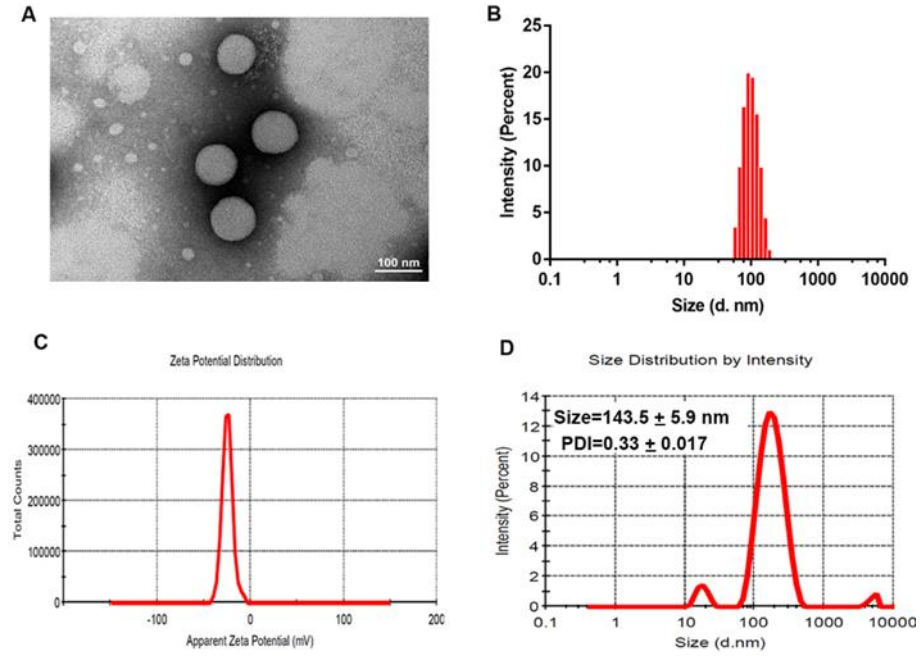

Figure S1. The characterization of non-PEGylated CPG/PPa NPs. (A) TEM image. (B) Intensity distribution profile of size. (C) The zeta potential of non-PEGylated CPG/PPa NPs. (D) Intensity distribution of non-PEGylated CPG/PPa NPs after incubation with PBS including 10% FBS for 4 h.

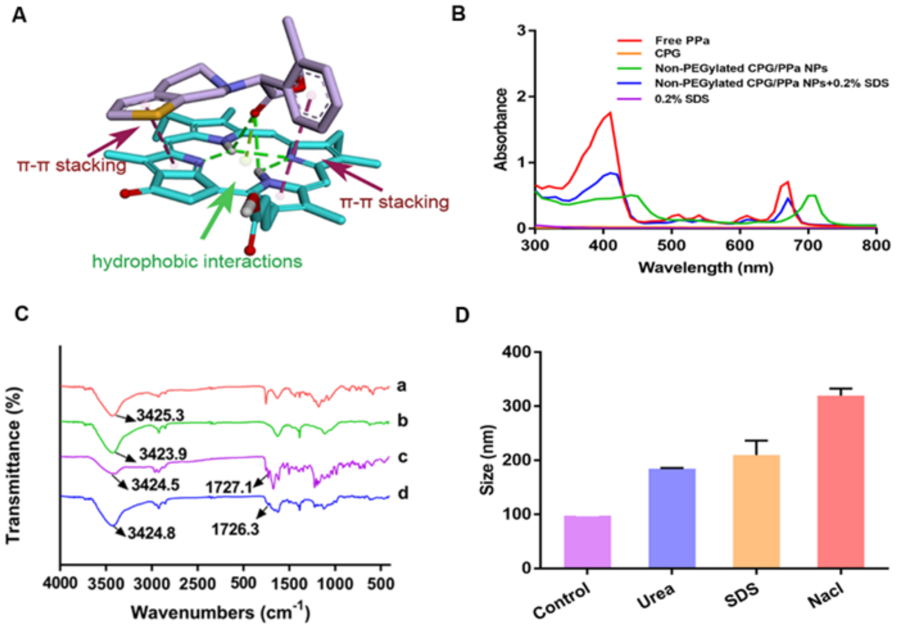

Figure S2. The co-assembly mechanism of non-PEGylated CPG/PPa NPs. (A) Molecular dynamics simulations of CPG and PPa molecules. (B) The UV absorption of free PPa, CPG, non-PEGylated CPG/PPa NPs, non-PEGylated CPG/PPa NPs+0.2% SDS, 0.2% SDS. (C) The FT-IR spectra of CPG (a), CPG/PPa NPs (without pegylation) (b), CPG/PPa mixture (c), and PPa (d). (D) The size change of non-PEGylated CPG/PPa NPs treated with urea, SDS and NaCl (200 mM).

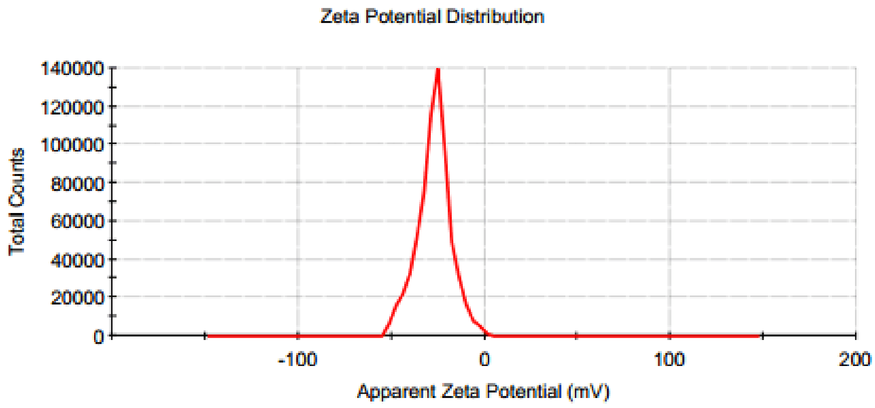

Figure S3. Zeta potential of CPG/PPa NPs.

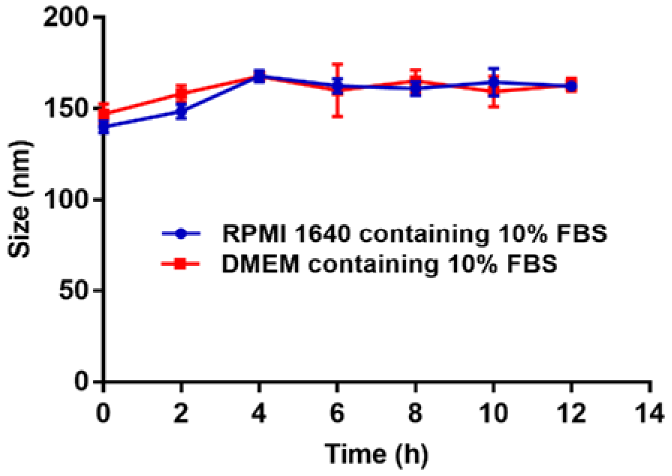

Figure S4. The stability of CPG/PPa NPs incubated in RPMI 1640 and DMEM medium containing 10% FBS for 12 h (n=3).

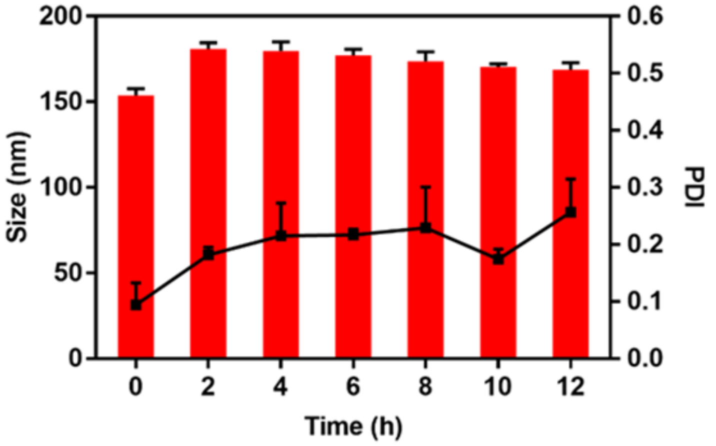

Figure S5. The colloidal stability of CPG/PPa NPs in plasma (n=3).

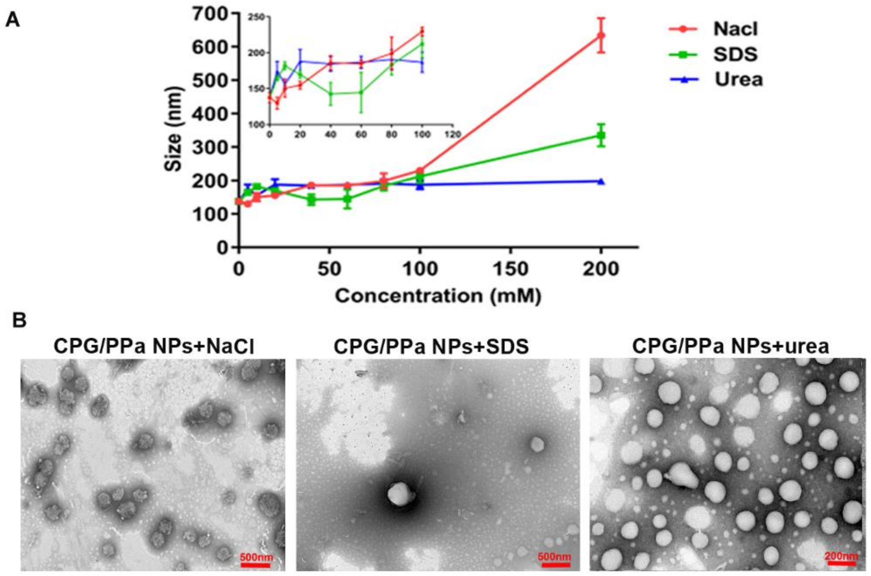

Figure S6. (A) The size changes of CPG/PPa NPs after treatment with different concentrations of NaCl, SDS, and urea, respectively. (B) The TEM images of CPG/PPa NPs treated with NaCl, SDS and urea (100 mM).

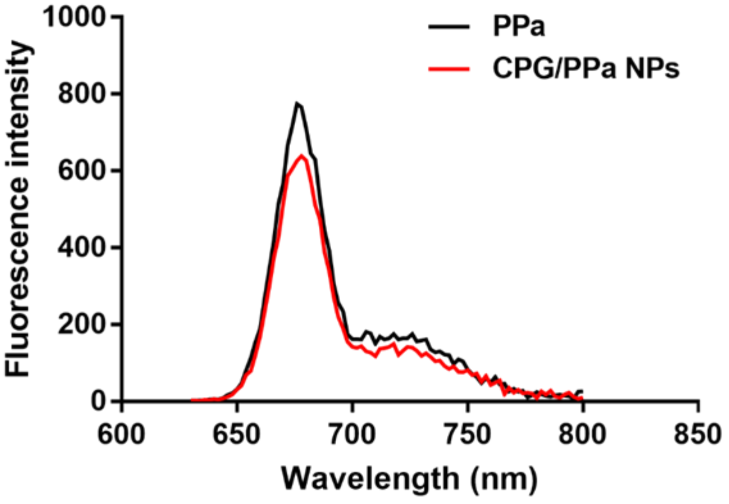

Figure S7. The PPa fluorescence spectra of free PPa and CPG/PPa NPs.

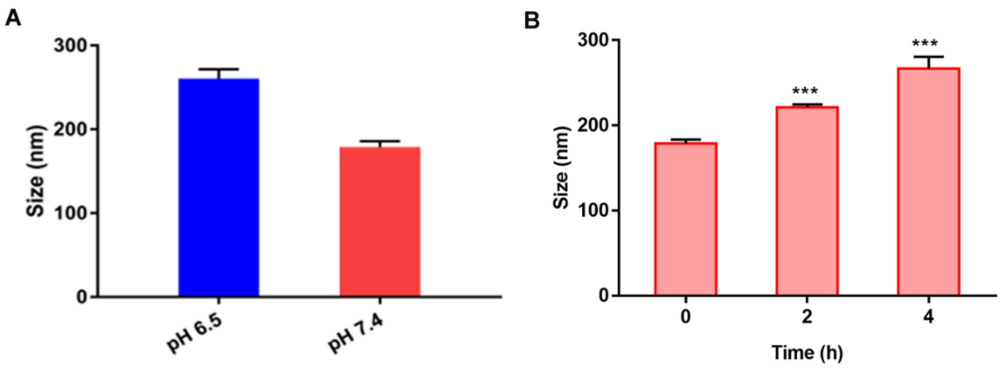

Figure S8. (A) The size changes of CPG/PPa NPs after treatment with different pH (n=3). (B) The stability of the CPG/PPa NPs in solution with pH 6.5 (n=3, ***P < 0.001).

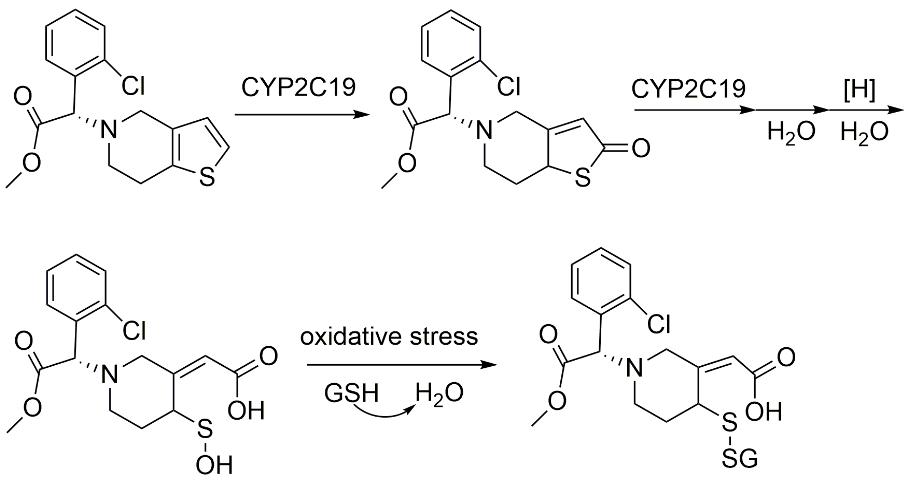

Figure S9. Metabolic pathway of CPG.

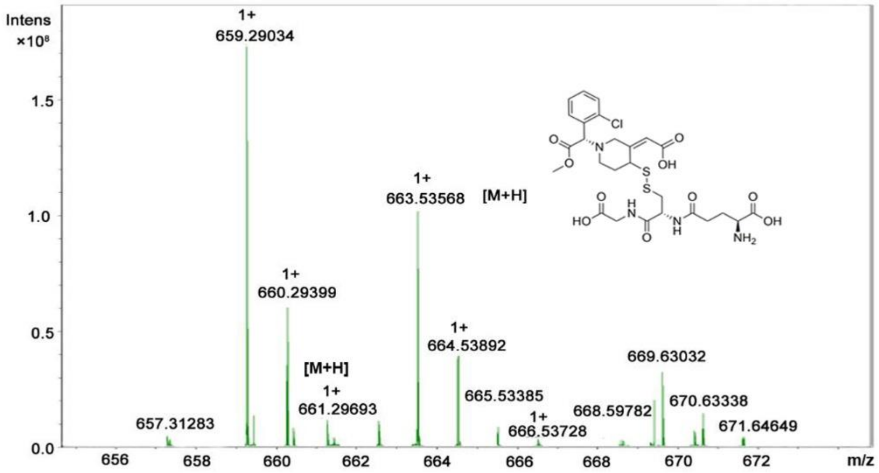

Figure S10. Mass spectrum of the GPG-SS-GSH.

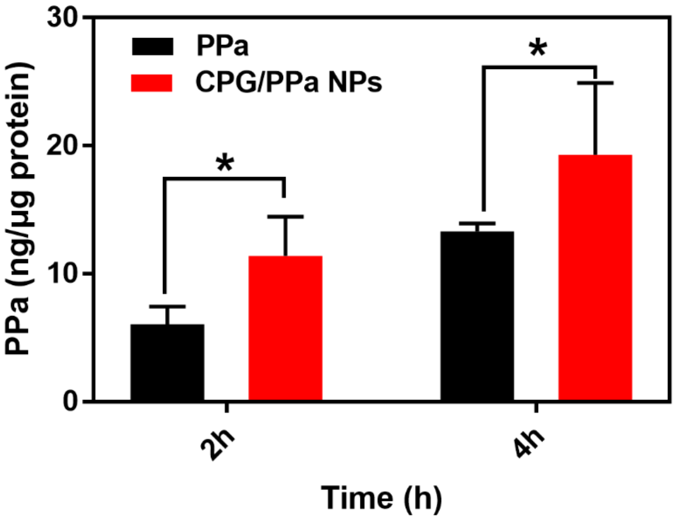

Figure S11. Quantitative analysis of cellular uptake of free PPa and CPG/PPa NPs at 2 h and 4 h.

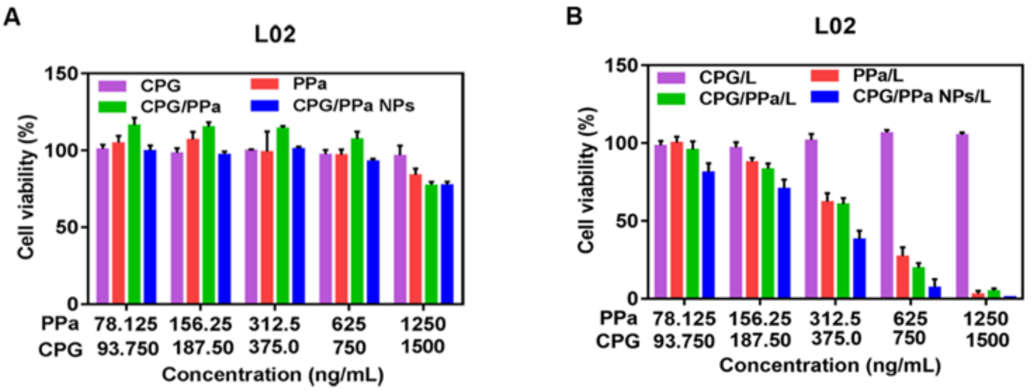

Figure S12. The cell viability of CPG, PPa, CPG/PPa mixture and CPG/PPa NPs against L02 cells with or without laser irradiation (n=3). (A) Without laser irradiation. (B) With irradiation (660nm, 30 mW/cm^2 for 2 min).

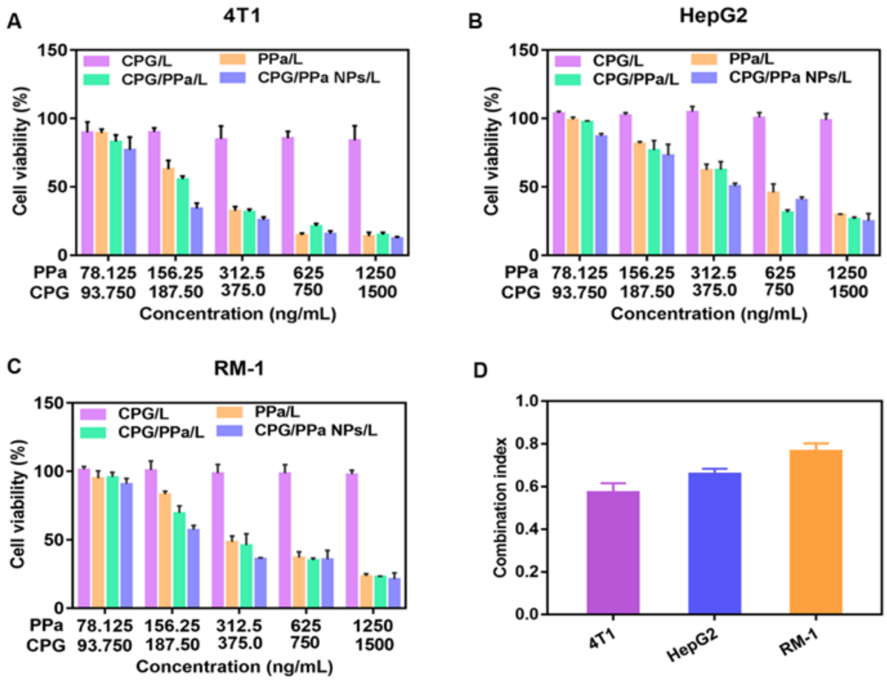

Figure S13. *In vitro* cell viability of CPG, PPa, CPG/PPa mixture and CPG/PPa NPs against 4T1, HepG2 and RM-1 cells incubated with GSH-OEt under laser irradiation (n=3). (A) 4T1 cells, (B) HepG2 cells, (C) RM-1 cells. (D) The combination index (50% inhibition) of CPG/PPa NPs in 4T1, HepG2 and RM-1 cells.

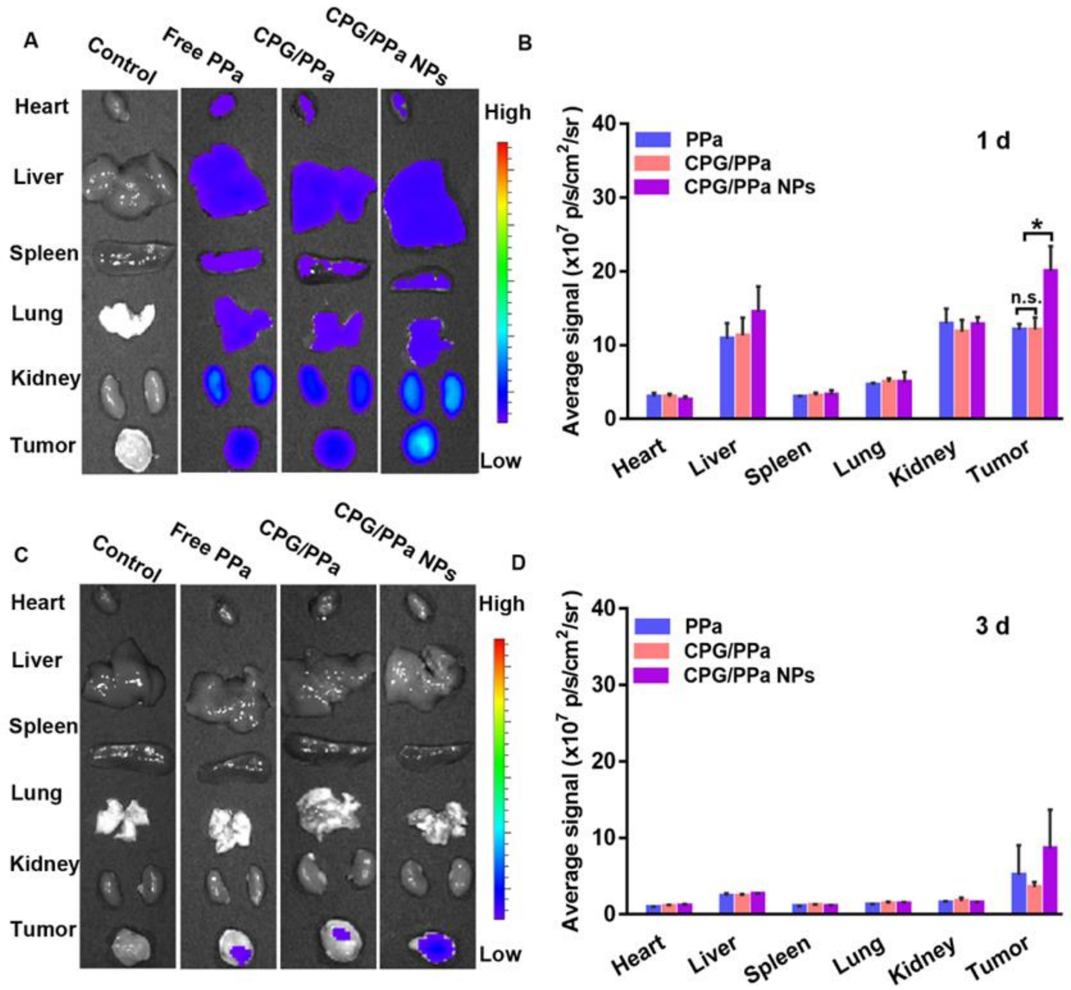

Figure S14. The biodistribution of PBS, free PPa, CPG/PPa and CPG/PPa NPs in 4T1 tumor-bearing BALB/c mice. (A) *In vitro* fluorescence imaging of major organs and tumors at 1 d, (B) Quantitative analysis average fluorescence intensity at 1 d; (C) *In vitro* fluorescence imaging of major organs and tumors at 3 d; (D) Quantitative analysis average fluorescence intensity at 3 d (n = 3, n.s. no significance, *P < 0.05,).

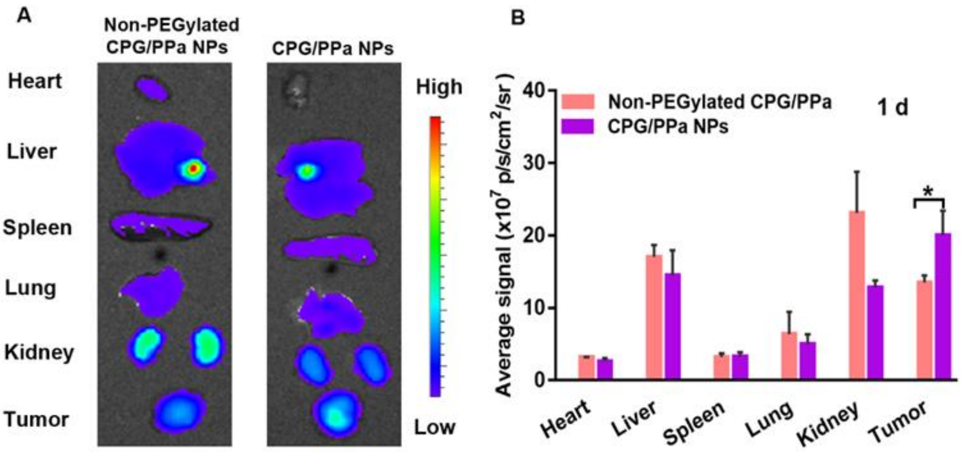

Figure S15. The biodistribution of non-PEGylated CPG/PPa NPs and CPG/PPa NPs in 4T1 tumor-bearing BALB/c mice at post 1 d administration. (A) *In vitro* fluorescence imaging of major organs and tumors, (B) Quantitative analysis average fluorescence intensity (n = 3, *P < 0.05).

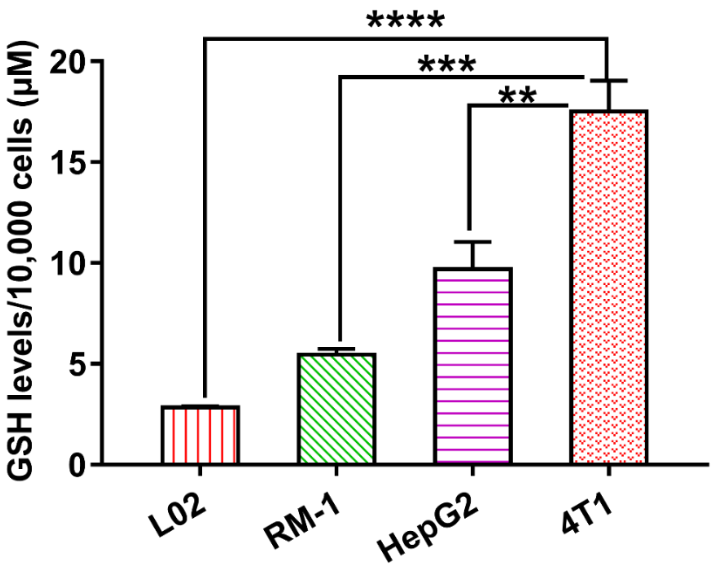

Figure S16. The GSH levels of L02, RM-1, HepG2, and 4T1.

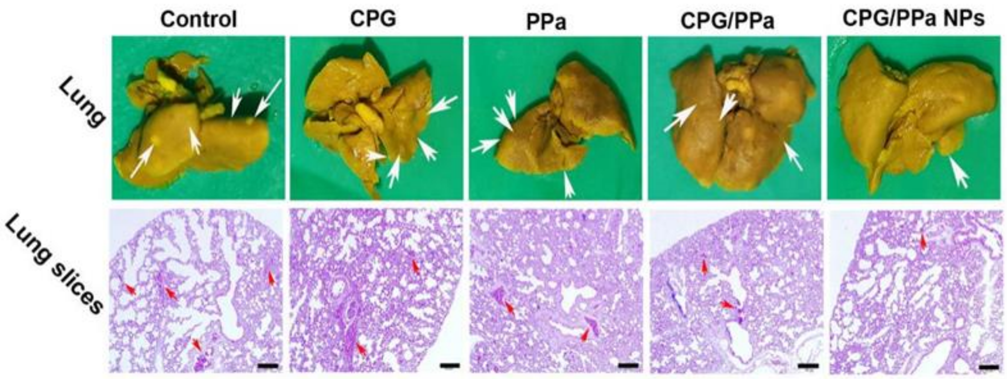

Figure S17. Images of picric acid staining of lungs and H&E staining of the lung slices prepared from different administration groups after treatments.

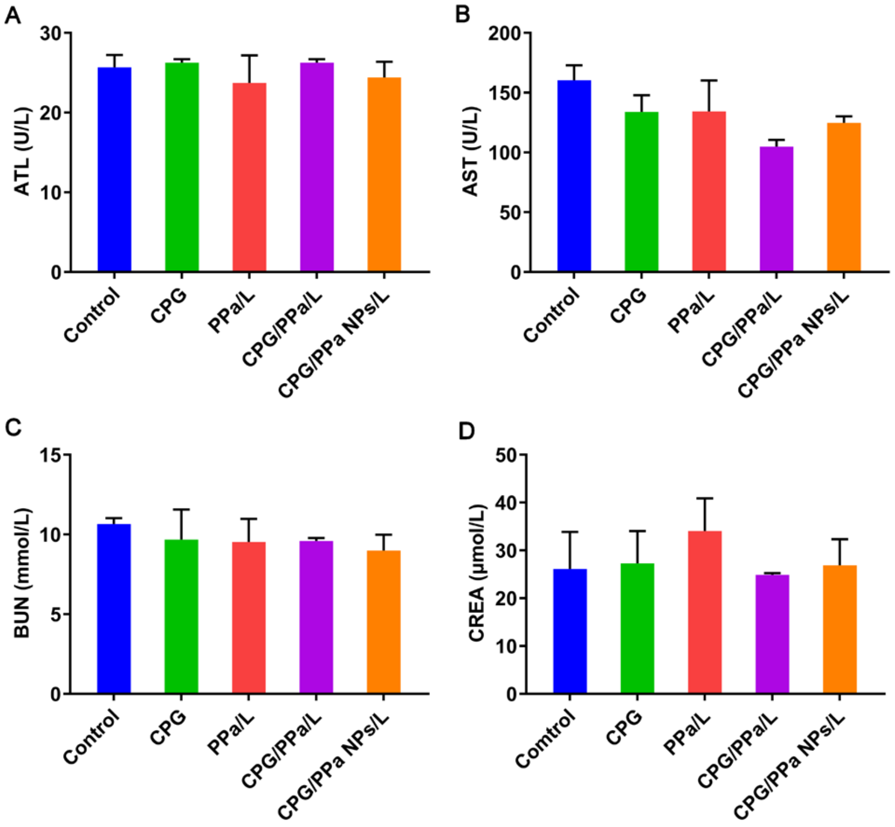

Figure S18. Hepatic and renal function parameters after the last treatment. (A) ALT: alanine aminotransferase; (B) AST: aspartate aminotransferase; (C) BUN: blood urea nitrogen; (D) CREA: creatinine. (n=3)

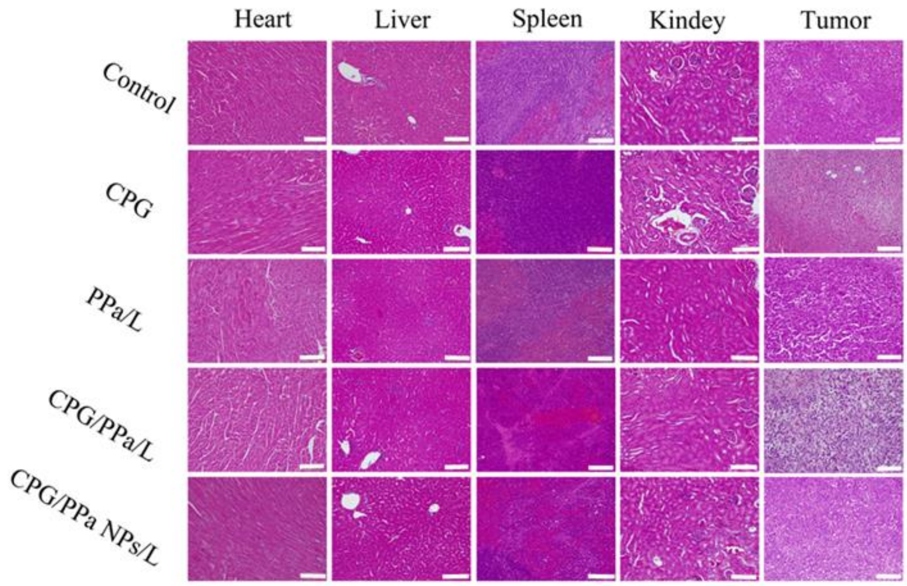

Figure S19. H&E staining of the major organs and tumors after treatments. Scale bar represents 100 μm.

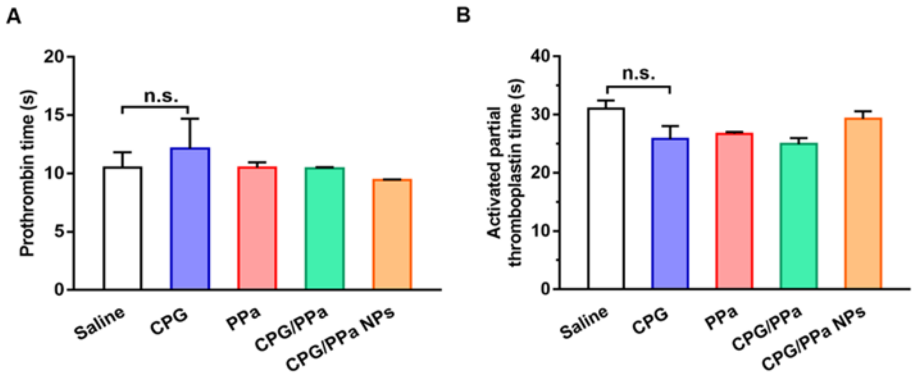

Figure S20. The coagulation indicators after last treatment. (A) Prothrombin time (PT). (B) Activated partial thromboplastin time (APTT).
